# Potential Mechanisms of MAP Kinase JNK’s Involvement in Modulating Cancer Cell Fate in a Cisplatin Concentration-Dependent Manner

**DOI:** 10.3390/ph19030509

**Published:** 2026-03-20

**Authors:** Monika Tenkutytė, Audronė V. Kalvelytė, Aurimas Stulpinas

**Affiliations:** 1 Apoptosis Research Group, Institute of Biochemistry, Life Sciences Center, Vilnius University, Saulėtekio av. 7, LT-10257 Vilnius, Lithuania; monikatenk@gmail.com (M.T.); audrone.kalvelyte@gmc.vu.lt (A.V.K.); 2 Department of Molecular Cell Biology, Institute of Biochemistry, Life Sciences Center, Vilnius University, Saulėtekio av. 7, LT-10257 Vilnius, Lithuania

**Keywords:** cancer, DNA damaging drugs, c-JUN N-terminal kinase (JNK), tumor suppressor p53, protein kinase AKT, histone H2AX, KRAS, reactive oxygen species (ROS)

## Abstract

**Background:** The combination of conventional drugs and inhibitors of signaling molecules is an effective strategy to increase cancer treatment efficacy and reduce drug doses to protect against their cytotoxic effects. Our research has shown the cisplatin concentration-dependent shift in the role of MAP kinase JNK from antiapoptotic to proapoptotic in non-small cell lung cancer A549 cells. Cell death/survival signaling molecules, tumor suppressor p53 and pro-survival protein kinase AKT were detected to be differently regulated by JNK inhibition at low vs. high cisplatin concentrations. Here, we further investigated the phenomenon and potential mechanisms of combined JNK inhibition and cisplatin treatment. **Methods**: Cell death in vitro was evaluated by MTT and Western blot assays after combined cisplatin and specific inhibitor treatment; two-way ANOVA was used for analysis. **Results:** JNK is differently involved in determining cellular sensitivity to different DNA-damaging drugs. There is no universal cell death induction mechanism originating from DNA damage through the involvement of JNK. The outcome of JNK inhibition also depends on the cell type. We found that there is an unusual reciprocal interaction between p53 and AKT in cisplatin-treated A549 cells, where p53 inhibits AKT, while AKT activates p53. In the case of cisplatin + JNK inhibitor SP600125, DNA damage and reactive oxygen species (ROS) contribute to cell death regulation in different ways. ROS exert opposite roles on cell fate-determining molecules p53 and AKT, and ROS act on p53 and AKT in opposite directions at low vs. high concentrations of cisplatin, combined or not with JNK inhibition. The differentially activated p53 in response to ROS (at low versus high concentrations of cisplatin, combined with JNK inhibitor) may be a molecular switch in the role of JNK from antiapoptotic to neutral/proapoptotic, and an executor of cell death. ROS is a possible threshold regulator that, together with an as-yet-unidentified factor, can differentially regulate p53. As a result, AKT phosphorylation and function are altered. The findings emphasize the importance of assessing the role of drug concentration when combining them with JNK inhibition when monitoring therapeutic efficacy and toxicity issues in personalized cancer treatment.

## 1. Introduction

The mechanisms of cancer drug action in determining cellular resistance and drug efficacy are being widely studied to identify downstream players, effectors, and potential targets, thereby creating new targeted drugs with better efficacy than existing ones, as well as new therapeutic strategies. Understanding the signal transduction networks of proliferation and cell death upon exposure to anticancer drugs is an essential way to identify new drug targets and biomarkers in cancer therapy. Manipulating the signals induced by chemotherapeutic drugs is a promising strategy for targeted cancer therapy.

Mitogen-activated protein kinase (MAPK) and PI3K/AKT signaling pathway molecules are involved in the regulation of cancer cell proliferation, differentiation, and death; therefore, they are potential targets for personalized therapy. They also participate in and regulate signal transduction of conventional drug-induced cell death and thus, their inhibition (or activation) might increase the efficacy of cancer treatment.

It is well known that, in addition to genetic rearrangements, non-genetic modulation of the cellular state can strongly influence drug responses. In response to treatment, the functionality of the aforementioned factors can determine cell fate; therefore, protein kinases MAPK and AKT are attractive targets to improve the efficacy of targeted or conventional chemotherapy.

Combination therapy is one of the most promising methods for cancer treatment, allowing us to solve the problems of drug effectiveness, resistance to drugs, and their toxic side effects. In the search for new combinations of well known anticancer agent cis-diamminedichloroplatinum (II) (cisplatin) in the treatment of lung cancer, we investigated stress-activated protein kinase JNK (c-Jun NH2-terminal protein kinase) as a potential target for cotreatment. JNK is a member of the MAPK family, activated in response to stresses and inflammatory signals, and it is involved in the regulation of a variety of cellular processes, such as apoptosis, cell proliferation, and differentiation.

It is generally accepted that JNK is the key transmitter of the apoptosis signal in cancer cells. However, there is increasing evidence for an opposite, anti-apoptotic role of JNK in regulating cancer cell survival. Studies using a variety of different cancer models point to JNK as a potential target to improve cancer therapy.

In our work, we used a combination of small molecule pharmacological inhibitors of the JNK signaling pathway with conventional therapeutic agent cisplatin, one of the most common and most effective chemotherapeutic drugs, which is widely used as an adjuvant therapy to treat various types of cancer, including lung cancer. However, due to the accompanying toxic side effects, as well as intrinsic, innate and acquired tumor cell resistance, its efficacy may be limited and therefore requires new approaches to application [1].

One of the drawbacks of conventional chemotherapy, including cisplatin, is cytotoxicity towards healthy cells. Side effects such as hearing loss, nephrotoxicity, acute kidney injury, etc., occur in cancer patients. This is usually addressed by reducing the chemotherapy dosage or delaying the treatment [2,3]. However, and paradoxically, it is known that low doses of chemotherapy can promote tumor growth, stimulate cell cycle progression rather than arresting or inhibiting cancer cell proliferation, and activate the ROS pathway [4]. Prolonged treatments may lead to chronic kidney disease and promote tumor angiogenesis and the emergence of resistance [3,5]. Therefore, combination treatments leading to increased cancer cell death might be the solution to the problems encountered using low doses of cisplatin.

In our previous works, we have shown that JNK inhibition at a low cisplatin concentration was highly effective in killing non-small-cell lung cancer A549 cells by inducing apoptosis. Different ATP-competitive inhibitors (SP600125, AS601245, bentamapimod, JNK2 inhibitor IX) and a peptide-based inhibitor XG-102, acting outside of JNK ATP binding pocket, resulted in efficient A549 cell viability reduction. The A549 cell line is known as lung carcinoma-derived, wild-type tumor suppressor p53-bearing, and KRAS G12S mutation-driven cancer cell line. The studies revealed that JNK plays a protective role at low cisplatin concentrations but an apoptosis-promoting or neutral role at high cisplatin concentrations in A549 cells. We have also identified the transcription factor p53 and the survival kinase AKT as potential targets of JNK pathway inhibition [6].

In this work, we aimed to determine the effect of JNK inhibition, a phenomenon that we demonstrated for cisplatin in A549 cells, on the efficacy of various other DNA-damaging platinum-based and platinum non-containing drugs, depending on their concentration. We also sought to clarify the dose-dependence of the role of JNK inhibition on the cisplatin response in other cancer cell lines, taking into account the expression of oncogenic KRAS in them.

It is generally accepted that the antitumor activity of cisplatin is mainly attributed to its binding to DNA, although the activated aqueous form of cisplatin can bind to a variety of targets, such as proteins, RNA and DNA molecules, membrane phospholipids, and microfilaments [7]. On the other hand, accumulating data indicates that the formation of cisplatin-DNA adducts, especially in non-dividing cells, is not enough to cause cellular toxicity. In addition to the cytotoxic effects associated with DNA damage, cisplatin generates large amounts of reactive oxygen species (ROS) in cells [8,9]. Thus, it is believed that DNA crosslinking and ROS generation are the main cytotoxic mechanisms induced by cisplatin.

Therefore, we continued drug combination studies to identify the potential mechanisms underlying the cisplatin concentration-dependent differential role of JNK in A549 cells, including the role of DNA damage and ROS signaling in response to JNK inhibition at low or high cisplatin concentrations. The relationship between previously identified signaling molecules p53 and AKT, involved in determining the role of JNK in cisplatin-induced signaling, was investigated.

## 2. Results

### 2.1. JNK Inhibition Effect on the Efficacy of Various DNA-Damaging Platinum-Containing and Non-Platinum Drugs, Depending on Their Concentration

We have shown that the role and efficacy of JNK inhibition in inducing cell death in lung cancer A549 cells depend on the cisplatin concentration. Combining JNK inhibition with low, sublethal concentrations of cisplatin is highly effective in inducing cell death, demonstrating a protective, antiapoptotic role of JNK, which shifts to a neutral or proapoptotic role with increasing cisplatin concentrations. In this article, a high concentration of the drug usually reduced the cellular viability by more than half in 72 h.

**Figure 1:**
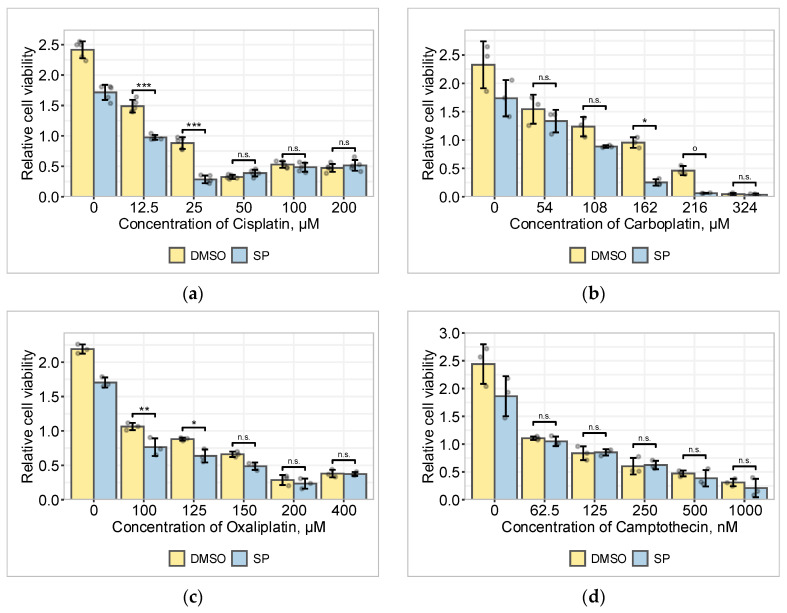

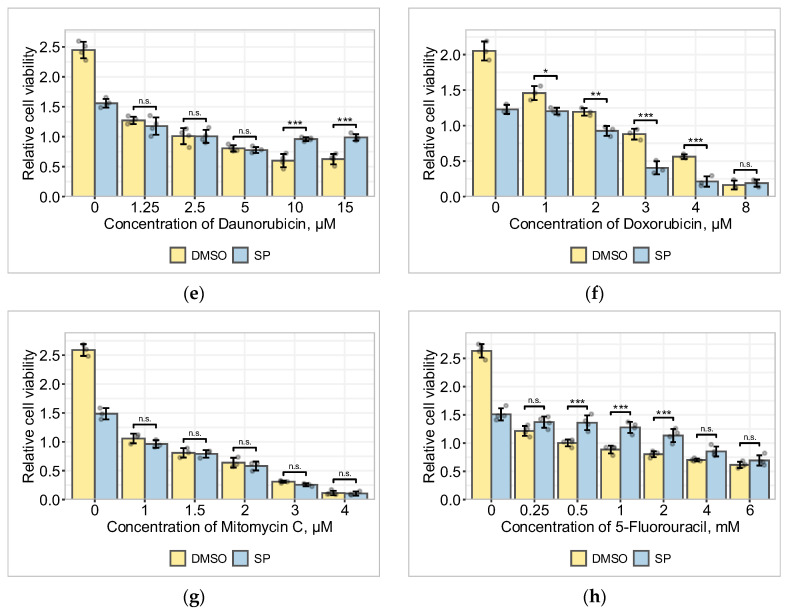
Effect of JNK inhibition in A549 cells treated with different chemotherapeutic drugs. (**a**) JNK inhibitor SP600125 (SP, 20 μM) significantly reduces cell viability when co-administered with a sublethal concentration of cisplatin (25 μM). (**b**) SP sharply reduces cell viability when co-administered with a sublethal concentration of carboplatin (162 μM). (**c**) SP slightly reduces cell viability when co-treated with a sublethal concentration of oxaliplatin (100–125 μM). (**d**) SP has no influence on cell viability when co-treated with camptothecin (62.5 nM–1 μM). (**e**) SP protects A549 cells from daunorubicin (10–15 μM). (**f**) SP reduces cell viability when co-treated with a sublethal concentration of doxorubicin (3 μM). (**g**) SP has no influence on cell viability when co-treated with Mitomycin C (1–4 μM). (**h**) SP protects A549 cells from 5-Fluorouracil (0.5–6 mM). Cell viability was measured by an MTT assay after 72 h of treatment and normalized to the initial viability before treatment, with this ratio (relative cell viability) being plotted on the *Y*-axis (see Methods). DMSO—vehicle control (0.1%). Statistical significance is indicated by asterisks (n.s.—non-significant; *—*p* < 0.05, **—*p* < 0.005, ***—*p* < 0.0005; more details in the Methods and Table 1).

In our further studies, we aimed to determine whether the change in the role of JNK is specific to cisplatin in A549 cells or whether other anticancer drugs also cause such an effect, i.e., whether the JNK inhibitor SP600125 (hereinafter—SP) causes the same killing effect in combination with other drugs at sublethal drug concentrations but not at higher drug concentrations (Figure 1a). Thus, we evaluated the cell viability in response to other anticancer drugs in combination with JNK inhibition.

We selected seven DNA damaging drugs commonly used in cancer therapy: platinum-based carboplatin and oxaliplatin, alkaloidal topoisomerase I inhibitor camptothecin, anthracyclines and topoisomerase II inhibitors doxorubicin and daunorubicin, antibiotic mitomycin C, and anti-metabolite 5-fluorouracil. In cytotoxicity studies, we determined sublethal and lethal drug concentrations and exposed A549 cells to a wide range of concentrations of these drugs in combination with SP (Figure 1b–h). To assess the effect of JNK inhibition on cell viability compared with cisplatin, we focused on sublethal drug concentrations.

The data presented in Figure 1 demonstrate the differential role of JNK inhibition on the viability of cells exposed to different DNA damage-inducing anticancer drugs.

A549 cell viability was significantly reduced by exposure to the JNK inhibitor SP at low cisplatin and carboplatin concentrations: 25 μM and 162 μM, respectively. The effect of carboplatin was most similar to that of cisplatin—at a sublethal carboplatin concentration of 162 μM, SP reduced the viability of A549 cells by approximately 70% (in the case of cisplatin, also about 70%) (Figure 1b).

The effect of oxaliplatin’s combination with JNK inhibition on cell death induction was weaker. At sublethal oxaliplatin concentrations of 100–125 μM, SP slightly reduced the viability of cells (Figure 1c).

JNK inhibition did not affect the effect of camptothecin over the wide range of drug concentrations tested (Figure 1d). Studies of JNK inhibition with anthracyclines, daunorubicin and doxorubicin, have shown opposing results. In the case of daunorubicin, JNK inhibition significantly protected the cells at cisplatin concentrations above the concentration, which reduced viability by 50% in 72 h. The data suggested that the proapoptotic function of JNK was evident over a wider range of daunorubicin concentrations (Figure 1e). At the same time, the inhibition of JNK in cells exposed to different concentrations of doxorubicin promoted cell death (Figure 1f).

The combination of mitomycin C, a DNA crosslinking antineoplastic antibiotic, with JNK inhibitor SP showed an effect that was very similar to that of camptothecin—JNK inhibition did not affect mitomycin C-induced A549 cell death (Figure 1g). Meanwhile, in the case of the anti-metabolite 5-fluorouracil (5-FU), JNK played a proapoptotic role, as its inhibition significantly protected cells from death over a wide range of drug concentrations used (Figure 1h).

Based on the above data, it can be concluded that JNK function in A549 cells varies depending on the chemotherapeutic drug used. The role and the efficacy of JNK inhibition using SP depend on the concentration of the drug it is combined with. In our studies, JNK inhibition significantly increased the death of A549 cells only when exposed to low sublethal concentrations of cisplatin or carboplatin. Therefore, we termed the phenomenon of the effective sensitization of cancer cells to only low concentrations of chemotherapeutic drugs by using SP600125, along with the reversal of this effect to neutral/opposite at high drug concentrations, as the “SP effect.”

Thus, anticancer drugs that cause DNA damage did not share a common mechanism that would lead to a switch in the role of JNK from antiapoptotic to neutral or proapoptotic; this property was a characteristic of cisplatin, the platinum-containing chemotherapeutic compound.

### 2.2. JNK Inhibition Studies in the Context of Oncogenic RAS

Next, in order to assess the possible role of mutated RAS in our identified phenomenon—the “SP effect”—we aimed to investigate the effect of RAS inhibition on A549 cell viability after treatment with a combination of cisplatin and SP, and also to determine whether the effect of cisplatin + SP is observed in other cancer cell lines with *RAS* mutations. It is known that RAS activating mutations are common in various cancers and they constitute about a quarter of known cancer-associated mutations, as well as conferring resistance to chemotherapy [10].

#### 2.2.1. RAS Inhibition Effect on A549 Cell Viability After Cisplatin Treatment

Since A549 cells harbor a *KRAS* driver mutation resulting in a constantly active KRAS (G12S) protein, which may be involved in JNK signaling [11], we investigated whether active KRAS is the causative factor of the “SP effect” in A549 cells. If it was, the “SP effect” would not occur in KRAS-suppressed cells.

**Figure 2:**
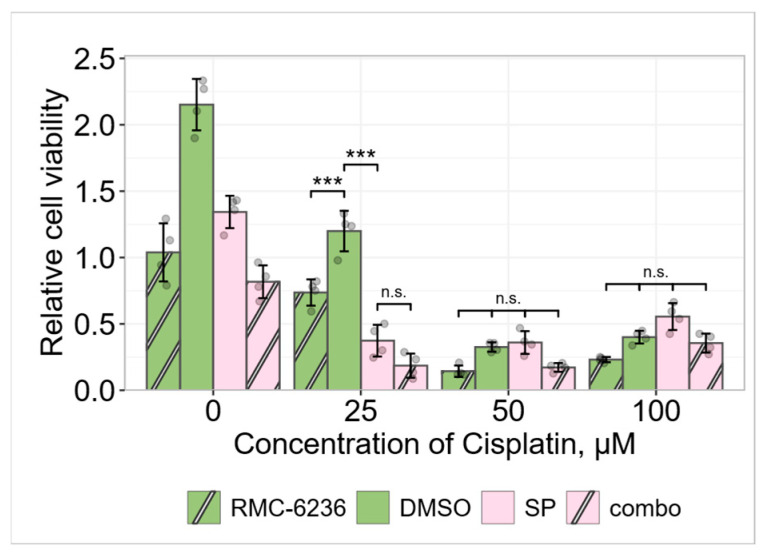
Effect of JNK and KRAS inhibition in cisplatin-treated A549 cells. Cell viability was measured via MTT assay after 72 h of treatment and normalized by initial viability before treatment. DMSO—vehicle control (0.2%), SP—SP600125 (20 μM), RMC-6236 (1 μM), and combo—SP + RMC. Statistical significance is indicated by asterisks (n.s.—non-significant; ***—*p* < 0.0005).

In our experiments, the novel RAS-ON inhibitor RMC-6236, which exhibits RAS inhibition of the active (GTP-bound) state of both mutant and wild-type variants of canonical RAS isoforms [12], alone and in combination with SP reduced A549 cell viability throughout the entire cisplatin concentration range used (Figure 2). Combining JNK and RAS inhibition revealed an additive pattern of cell viability reduction, meaning that SP and RMC-6236 compromise A549 cell viability through different pathways. Since KRAS inhibition did not abolish the “SP effect”, we can conclude that persistently activated KRAS is not necessarily the primary cause of the “SP effect”.

#### 2.2.2. JNK Inhibition Studies in Cisplatin-Treated Cancer Cell Lines in the Context of Oncogenic RAS

**Figure 3:**
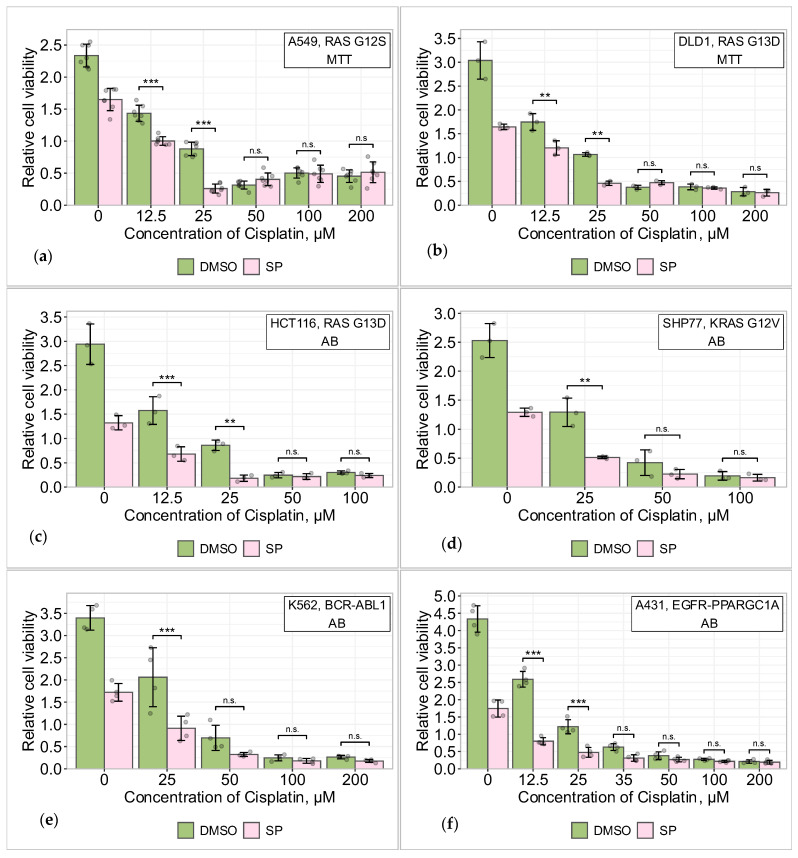
Effect of JNK inhibition in different *KRAS* mutation status-bearing cisplatin-treated cells. Boxes on the top right of the graphs indicate the cell line name, known driver mutation, and the cell viability assay method—MTT or Alamar Blue (AB). Cell viability was measured after 72 h of treatment and normalized by initial viability before treatment. DMSO—vehicle control (0.1%) and SP—SP600125 (20 μM). Statistical significance is indicated by asterisks (n.s.—non-significant; **—*p* < 0.005, and ***—*p* < 0.0005).

To determine whether the “SP effect” is specific to *KRAS*-mutated cells or is fairly universal across many cancer cells, regardless of whether they harbor a *KRAS* mutation, we tested several cancer cell lines with and without a *KRAS* mutation. In previous studies, we did not detect the “SP effect” in primary lung cancer cell lines with unexamined *KRAS* mutation status at low cisplatin concentrations [6]. In this work, we tested five established commercial cell lines: three harboring a *KRAS* mutation (DLD1, HCT116, SHP77) and two harboring other driver mutations (K562 and A431). All tested cell lines exposed to the JNK inhibitor SP exhibited some level of sensitization to cisplatin treatment at low/sublethal doses, but no such effect was observed at high doses (Figure 3).

Combined cisplatin and SP treatment in A549, DLD1, HCT116, SHP77, K562, and A431 cells resulted in a decrease in cell viability to varying degrees at low/sublethal cisplatin concentrations, suggesting that JNK functions as a protective antiapoptotic protein in these cases. A more pronounced “SP effect” was seen in A549, DLD1, and HCT116 cell lines carrying *KRAS* mutations than in cells without them.

However, based on these and RAS inhibition data, we cannot currently state that JNK-mediated sensitization to low concentrations of cisplatin depends on the *KRAS* mutational status of the cells. Cell lines with different *KRAS* mutational status need to be further investigated by repairing the known mutations, followed by Western blot analysis, to definitively state whether the “SP effect” is caused by KRAS activation.

In summary, the “SP effect” is not unique to A549 cells; it is observed in different types of cancer cell lines harboring mutated *KRAS*, but we lack further evidence to declare that it could be universal to all cancer cell lines.

### 2.3. Ataxia Telangiectasia-Mutated (ATM) Involvement: Effect of ATM Inhibition on Cell Viability and Histone H2AX Phosphorylation

Our data, presented above, showed an antiapoptotic role of JNK in lung cancer A549 cells at low concentrations of cisplatin. When the concentration of cisplatin was increased, this antiapoptotic effect of JNK changed to neutral or proapoptotic for the cells tested. In order to clarify the reasons for this phenomenon and possible mechanisms, we performed experiments to assess the involvement of DNA damage in determining JNK dependency on cisplatin concentration in cell fate regulation.

First, we performed biochemical assays (protein electrophoresis and subsequent Western blot) by using the biomarker of DNA damage—S139 phosphorylation of H2AX. ATM protein kinase is known as a DNA damage sensor and central regulator of the DNA damage response. Upon DNA damage, a component of the histone octamer in nucleosomes H2AX is phosphorylated at serine-139 by the protein kinase ATM.

**Figure 4:**
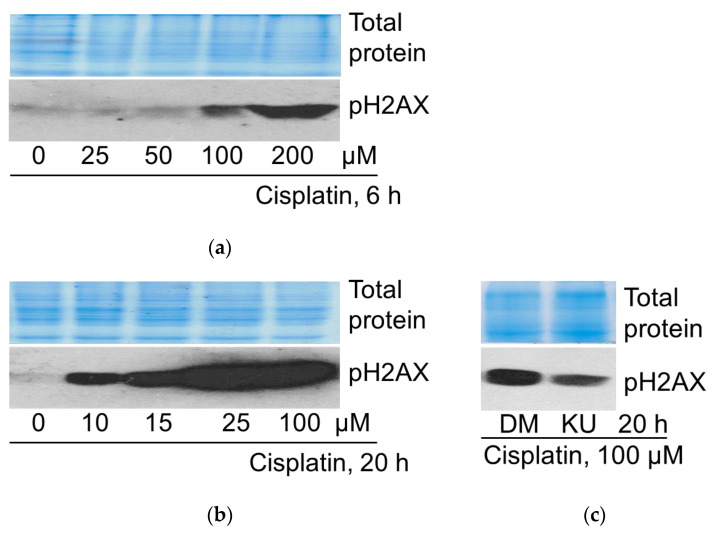
Induction of H2AX phosphorylation by cisplatin. (**a**) High concentration of cisplatin induces H2AX phosphorylation as early as 6 h after the treatment. (**b**) After 20 h, even very low concentrations of cisplatin induce H2AX phosphorylation. (**c**) ATM inhibitor reduces H2AX phosphorylation. DM—vehicle control DMSO (0.2%) and KU—KU60019 (5 μM). Representative Western blots are shown. Total protein stained with Coomassie dye in polyacrylamide gels serve as loading controls.

The results show that cisplatin (low and high concentrations) induced concentration-dependent phosphorylation of histone H2AX (Figure 4). High concentrations of cisplatin (100 and 200 μM) activated H2AX phosphorylation as early as 6 h after the treatment (Figure 4a), whereas the sublethal concentration of cisplatin (25 μM) only at 20 h. Notably, much lower concentrations (10 and 15 μM) also induced H2AX phosphorylation at 20 h (Figure 4b). H2AX phosphorylation confirmed DNA damage in cisplatin-treated cells. ATM inhibitor KU60019 reduced the specific H2AX S139 phosphorylation, validating ATM as the responsible protein kinase (Figure 4c).

We further performed cell viability assays to explore the role of DNA-damage-sensing protein kinase ATM (“ataxia telangiectasia mutated”) in A549 cell fate regulation. ATM kinase is closely related to phosphoinositide 3-kinase and thus is known to participate in survival signaling. For this purpose, cells were treated with KU60019 (hereinafter KU), a selective inhibitor of ATM, at either a low, subtoxic (low, 25 μM), or fourfold higher (high, 100 μM) cell-killing concentration of cisplatin, with or without the JNK inhibitor SP.

**Figure 5:**
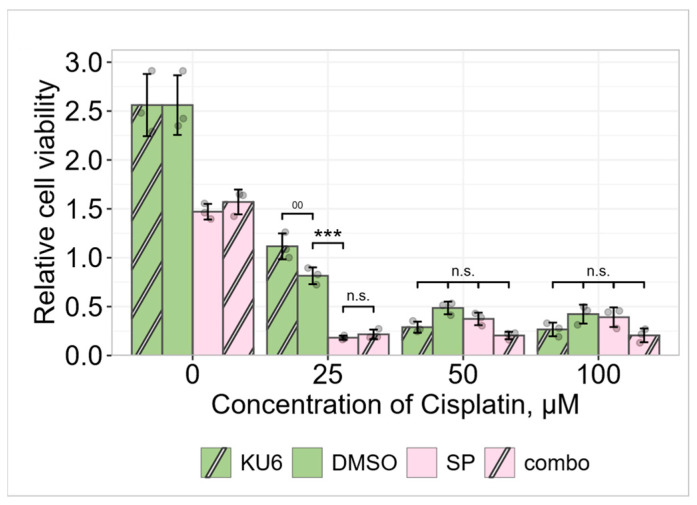
Effects of ATM inhibition on cisplatin-treated A549 cell viability. Cell viability was measured via MTT assay after 72 h of treatment and normalized to baseline viability before treatment. DMSO—vehicle control (0.2%), SP—SP600125 (20 μM), KU—KU60019 (5 μM), and combo—SP + KU. Statistical significance is indicated by asterisks (n.s.—non-significant; ***—*p* < 0.0005; more details in the Methods and Table 1).

Using KU, we showed that ATM inhibition protected A549 cells against cisplatin-induced cell death at a low cisplatin concentration without the JNK inhibitor SP, and only slightly in the presence of SP—in this case, the role of ATM kinase was proapoptotic. Conversely, at high cisplatin concentrations, KU reduced cell viability (Figure 5).

These studies showed that the DNA damage sensor protein kinase ATM differentially regulated A549 cell viability at low and high cisplatin concentrations.

### 2.4. ROS Involvement: Effects of ROS Scavengers on Cell Viability and Histone H2AX Phosphorylation

**Figure 6:**
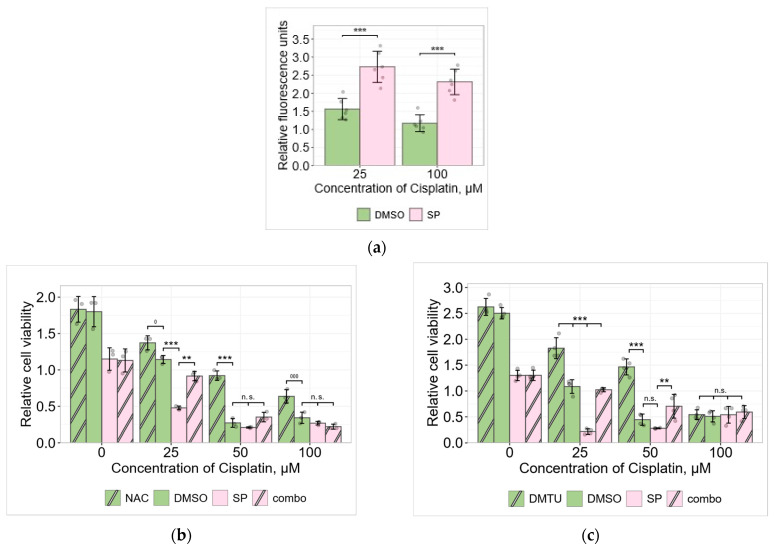
Reactive oxygen species in cisplatin- and SP-treated cell viability. (**a**) JNK inhibitor SP600125 increases ROS-sensitive fluorescence in A549 cells treated with cisplatin. Representative experiment is shown. *N* = 6 (technical replicates) for each group, *p* = 0.000420 for 25 μM cisplatin, and *p* = 0.000116 for 100 μM cisplatin. DMSO—vehicle control (0.1%) and SP—JNK inhibitor SP600125. (**b**) Pretreatment with N-acetyl cysteine (NAC, 2 mM) increases A549 cell viability. (**c**) Pretreatment with N,N′-dimethylthiourea (DMTU, 1 mM) increases A549 cell viability at low concentrations. Cell viability was measured via MTT assay after 72 h of treatment and normalized to baseline viability before treatment. DMSO—vehicle control (0.2%), SP—SP600125 (20 μM), and combo—SP + antioxidant. Statistical significance is indicated by asterisks (n.s.—non-significant; **—*p* < 0.005, and ***—*p* < 0.0005; more details in the Methods and Table 1).

Beyond nuclear DNA damage, cisplatin generates a significant amount of reactive oxygen species (ROS) inside the cells. ROS can participate in the activation of various intracellular signaling molecules involved in the regulation of cell viability and death. Cisplatin-induced ROS may cause additional DNA damage as well [13]. Our studies showed that JNK inhibition increased ROS-sensitive probe H2DCFDA fluorescence in our system (Figure 6). Therefore, to clarify the possible role of ROS in our model system, we performed experiments using the antioxidant N–acetylcysteine (NAC, 2 mM). The data provided in Figure 6b shows that NAC protected cells from death when exposed to both low and high concentrations of cisplatin (therefore, ROS promoted cell death), but not at a high cisplatin concentration when in combination with SP.

Thus, we have demonstrated the protective role of the antioxidant NAC in A549 cells exposed to low concentrations of cisplatin. However, it is known that in addition to its antioxidant effects, NAC can also directly bind cisplatin in the medium reducing cisplatin reactivity, which may contribute to its protective effects [14]. Therefore, to confirm the role of ROS in our system, we performed experiments using another ROS scavenger, N,N′-dimethylthiourea (DMTU, 1 mM). Cisplatin, among other ROS, induces the accumulation of hydroxyl radicals (•OH). DMTU acts as an antioxidant by scavenging hydroxyl radicals and blocking their production [9,15].

Cell viability studies showed that, as in the case of NAC, DMTU inhibited cell death in cells exposed to low cisplatin concentrations (Figure 6c). Thus, it can be stated that ROS are involved in inducing cell death at low cisplatin concentrations, both in the presence and absence of the JNK inhibitor SP600125.

**Figure 7:**
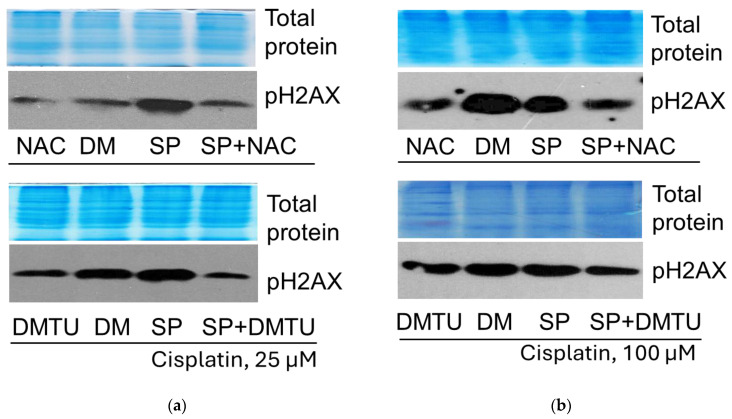
Antioxidants decrease H2AX phosphorylation. (**a**) In A549 cells treated with 25 μM cisplatin (both alone and in combination with the JNK inhibitor SP600125), H2AX phosphorylation is decreased after addition of the antioxidant NAC (2 mM) or DMTU (1 mM). (**b**) ROS scavengers NAC or DMTU reduce H2AX phosphorylation in A549 cells exposed to 100 μM cisplatin (both alone and in combination with the JNK inhibitor SP), just as in (**a**). JNK inhibitor alone increases H2AX phosphorylation at 25 μM cisplatin, in contrast to 100 μM cisplatin. DM—vehicle control DMSO (0.1%) and SP—SP600125 (20 μM); treatment duration—20 h. Representative Western blots are shown. Total protein stained with Coomassie dye in polyacrylamide gels serve as loading controls.

H2AX phosphorylation studies revealed that JNK inhibition (20 μM SP600125) increased H2AX phosphorylation at a “low” cisplatin concentration but decreased at “high” (Figure 7). At the same time, ROS-scavenging antioxidants inhibited H2AX phosphorylation in cells exposed to both 25 μM cisplatin and 100 μM cisplatin (both alone and in combination with the JNK inhibitor SP) (Figure 7), showing a possible link between ROS and DNA damage sensor ATM.

### 2.5. ATM Inhibition-Mediated Regulation of p53 and AKT

We next investigated the dependence of signaling molecules—the tumor suppressor transcription factor p53 and the pro-survival protein kinase AKT—on DNA damage and ROS induced in cells by cisplatin with or without JNK inhibition.

In a previous study, we have shown that levels of activated p53 and AKT in cisplatin-treated cells after cotreatment with the JNK inhibitor SP correlated with their role in regulating cell death, i.e., at a low cisplatin concentration, SP increased p53 protein expression and phosphorylation, decreased AKT phosphorylation, and sensitized the cells, while at a high cisplatin concentration SP decreased p53 protein expression and phosphorylation, increased AKT phosphorylation, and resulted in cisplatin resistance. Hence, we proposed these two molecules as signaling proteins mediating the outcome of JNK inhibition in A549 cells exposed to different concentrations of cisplatin. Using the p53 activator nutlin-3a and the AKT kinase inhibitor capivasertib, we demonstrated the proapoptotic action of these agents in our model system, as published previously [6]. Reduction in A549 cell viability when treated with AKT inhibitor VIII (10 μM) at all cisplatin concentrations tested, either alone or in combination with JNK inhibitor SP, additionally contributed to the findings (unpublished data).

#### 2.5.1. p53 Expression

**Figure 8:**
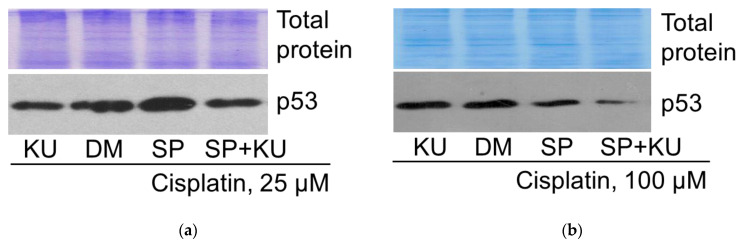
Effect of ATM kinase inhibition on p53 expression. (**a**) In A549 cells treated with 25 μM cisplatin (both alone and in combination with the JNK inhibitor SP600125), p53 expression is decreased after the addition of the ATM inhibitor KU60019 (KU, 5 μM). (**b**) In A549 cells exposed to 100 μM cisplatin and JNK inhibitor SP600125, ATM inhibitor KU decreases p53 expression. DM—vehicle control DMSO (0.2%), SP—SP600125 (20 μM); treatment duration—20 h. Representative Western blots are shown. Total protein stained with Coomassie dye in polyacrylamide gels serve as loading controls.

As shown previously, JNK inhibitor SP activated tumor suppressor p53 expression in 25 μM cisplatin-treated A549 cells. Further examinations showed that ATM kinase inhibitor KU decreased p53 expression in A549 cells treated with 25 μM cisplatin (both alone and in combination with the JNK inhibitor SP600125) (Figure 8a). In 100 μM cisplatin-treated A549 cells, contrary to 25 μM cisplatin, JNK inhibitor SP decreased tumor suppressor p53 expression. In this case, just like in the case of cisplatin 25 μM, ATM kinase inhibitor KU also reduced p53 expression (both alone and in combination with the JNK inhibitor SP600125) (Figure 8b). Thus, the effect of the ATM inhibition is the same at both 25 and 100 μM cisplatin concentrations—it reduces p53 expression in all cases studied.

#### 2.5.2. AKT Phosphorylation

**Figure 9:**
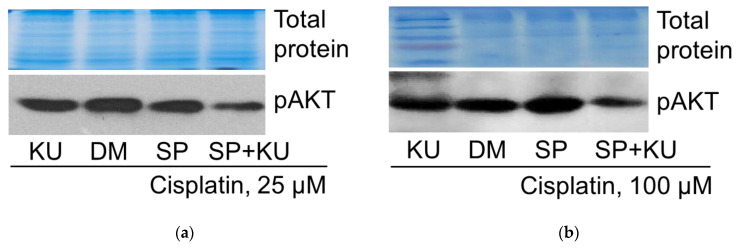
**Effect of ATM inhibition on protein kinase AKT phosphorylation.** (**a**) In A549 cells treated with 25 μM cisplatin (both alone and in combination with the JNK inhibitor SP600125), AKT phosphorylation is decreased after the addition of the ATM inhibitor KU60019 (KU, 5 μM). (**b**) In A549 cells exposed to 100 μM cisplatin and JNK inhibitor SP600125, ATM inhibitor KU decreases AKT phosphorylation. DM—vehicle control DMSO (0.2%) and SP—SP600125 (20 μM); treatment duration—20 h. Representative Western blots are shown. Total protein stained with Coomassie dye in polyacrylamide gels serve as loading controls.

As shown previously and above, JNK inhibitor SP reduced pro-survival kinase AKT phosphorylation (T308) in 25 μM cisplatin-treated A549 cells. This study revealed that exposure of cells to the ATM kinase inhibitor KU reduced AKT phosphorylation in A549 cells exposed to 25 μM cisplatin (both alone and in combination with the JNK inhibitor SP600125) (Figure 9a). The same was observed at a 100 μM cisplatin concentration (Figure 9b).

These results indicate that protein kinase ATM regulates AKT phosphorylation in the same manner at high and low concentrations of cisplatin, both alone and in combination with SP.

### 2.6. Different Regulation of p53 and AKT by ROS

#### 2.6.1. p53 Expression

**Figure 10:**
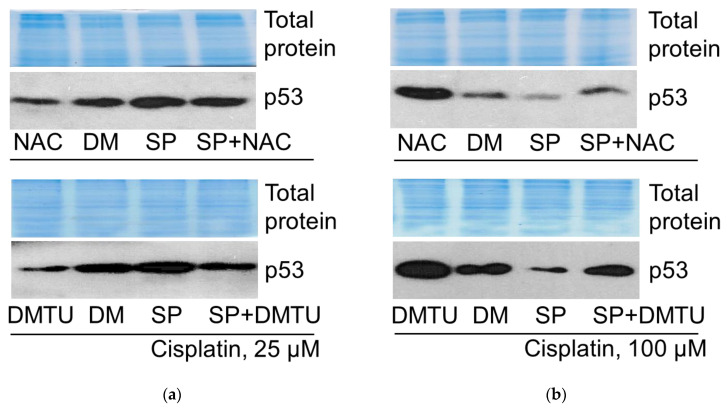
Opposite effect of antioxidants on p53 level in A549 cells treated with low and high cisplatin concentrations. (**a**) At a 25 μM cisplatin concentration, both antioxidants, N-acetylcysteine (NAC, 2 mM) and N,N′-dimethylthiourea (DMTU, 1 mM), decrease p53 levels in A549 cells, both alone and in combination with JNK inhibitor SP600125. (**b**) At a 100 μM cisplatin concentration, both antioxidants and their combinations with JNK inhibitor SP600125 have an opposite effect: they increase p53 levels in A549 cells. DM—vehicle control DMSO (0.2%), SP—SP600125 (20 μM) and treatment duration—20 h. Representative Western blots are shown. Total protein stained with Coomassie dye in polyacrylamide gels serve as loading controls.

Next, we aimed to determine whether ROS were involved in the regulation of p53 expression in cisplatin- and SP-treated A549 cells. The changes in p53 protein amount were monitored via Western blot. As we reported in our previous study [6], the JNK inhibitor SP activated tumor suppressor p53 expression in 25 μM cisplatin-treated A549 cells, whereas it decreased the p53 protein level in cells treated with 100 μM cisplatin. Data presented in Figure 10 confirm these findings. In order to assess the involvement of ROS in p53 protein expression regulation, we analyzed the impact of antioxidants on the p53 protein amount, using the Western blot method. ROS scavengers NAC (N-Acetyl-L Cysteine) and N,N′-dimethylthiourea (DMTU) were used either with or without SP in cisplatin-treated cells. p53 expression decreased after exposure to the antioxidants in A549 cells treated with 25 μM (both alone and in combination with the JNK inhibitor SP600125). However, p53 expression increased after exposure to the antioxidants in cells treated with 100 μM cisplatin (both alone and in combination with the JNK inhibitor SP600125) (Figure 10). Thus, ROS differently regulate p53, depending on cisplatin concentration.

#### 2.6.2. AKT Phosphorylation

**Figure 11:**
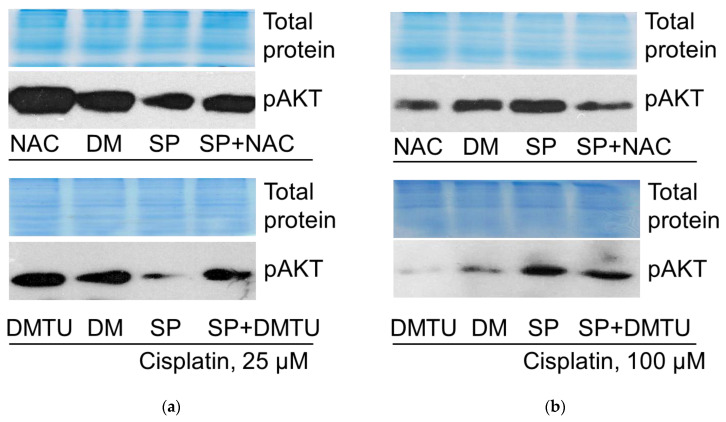
Opposite effect of antioxidants on AKT phosphorylation in A549 cells treated with low and high cisplatin concentrations. (**a**) At a 25 μM cisplatin concentration, both antioxidants, N-acetylcysteine (NAC, 2 mM) and N,N′-dimethylthiourea (DMTU, 1 mM) as well as their combinations with JNK inhibitor SP600125, increase AKT phosphorylation in A549 cells. (**b**) At a 100 μM cisplatin concentration, both antioxidants and their combinations with JNK inhibitor SP600125 have an opposite effect: they decrease AKT phosphorylation in A549 cells. DM—vehicle control DMSO (0.2%), SP—SP600125 (20 μM), and treatment duration—20 h. Representative Western blots are shown. Total protein stained with Coomassie dye in polyacrylamide gels serve as loading controls.

In contrast to p53, opposite direction changes were seen in AKT phosphorylation. JNK inhibitor SP reduced the survival protein kinase AKT phosphorylation (at T308) in A549 cells treated with 25 μM cisplatin, but increased AKT phosphorylation in the case of 100 μM cisplatin (Figure 11). Accordingly, and in contrast to the effects on p53, antioxidants NAC and DMTU increased AKT phosphorylation in A549 cells exposed to 25 μM cisplatin (both alone and in combination with the JNK inhibitor SP600125), and decreased AKT phosphorylation in the case of 100 μM cisplatin, both with and without SP.

### 2.7. The Interplay of Protein Kinase AKT and Tumor Suppressor p53

The relationship between the signaling molecules AKT and p53 in JNK-regulated signal transduction at different cisplatin concentrations was investigated using their specific modulators: AKT inhibitor capivasertib (CAP) and AKT inhibitor VIII (AKTi), and p53 activator (MDM2 inhibitor) nutlin-3a (NUT). Capivasertib (AZD5363) is an approved targeted drug for breast cancer treatment, whereas AKTi is a preclinical inhibitor of protein kinase AKT (with higher affinity towards AKT1 and AKT2). Nutlin-3a inhibits ubiquitin ligase MDM2, which binds p53 and directs it for proteasomal degradation; therefore, NUT increases p53 stability and its accumulation in the cytoplasm.

**Figure 12:**
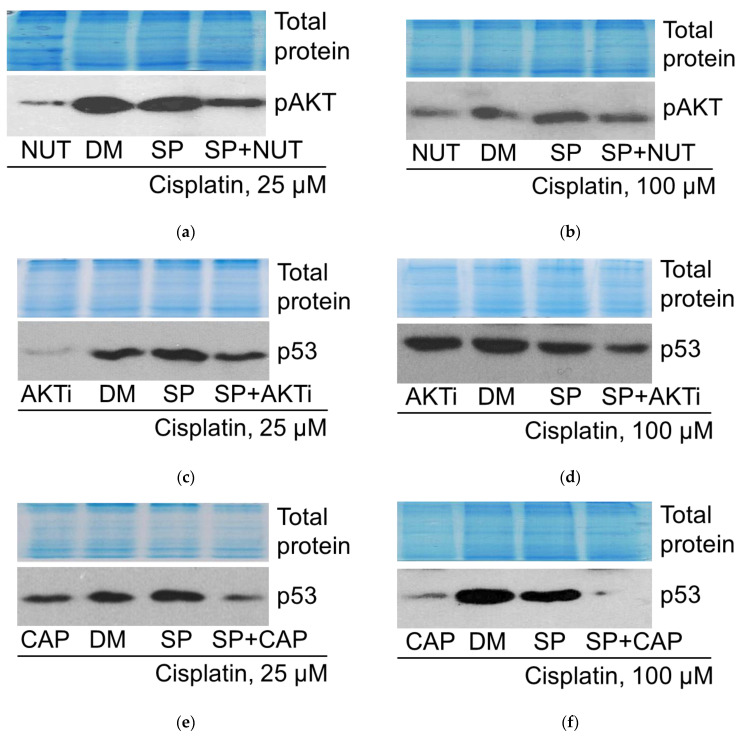
Interplay of tumor suppressor p53 and protein kinase AKT in cisplatin-treated A549 cells. (**a**) In A549 cells treated with 25 μM cisplatin (both alone and in combination with the JNK inhibitor SP), AKT phosphorylation is decreased after the addition of p53 activator nutlin-3a (NUT, 10 μM). (**b**) In A549 cells exposed to 100 μM cisplatin (both alone and in combination with the JNK inhibitor SP), AKT phosphorylation is decreased after the addition of p53 activator nutlin-3a. (**c**,**e**) In A549 cells treated with 25 μM cisplatin (both alone and in combination with the JNK inhibitor SP600125), p53 expression is decreased after the addition of AKT inhibitor VIII (AKTi, 10 μM) or capivasertib (CAP, 10 μM). (**d**,**f**) In A549 cells treated with 100 μM cisplatin (both alone and in combination with the JNK inhibitor SP600125), p53 expression is also decreased after the addition of AKT inhibitor VIII or capivasertib. DM—vehicle control DMSO (0.2%) and SP—SP600125 (20 μM); treatment duration—20 h. Representative Western blots are shown. Total protein stained with Coomassie dye in polyacrylamide gels serve as loading controls.

Investigating the role of p53 in regulating AKT activity, the phosphorylation status of AKT was analyzed in cisplatin-treated cells after treatment with the p53 activator nutlin-3a via Western blot. It is known that phosphorylation at T308 correlates with the kinasic activity of AKT and is linked to the RAS/PI3K/PDK1 signaling pathway [16]. The data presented in Figure 12a,b show that this AKT phosphorylation was diminished in A549 cells treated with both 25 μM and 100 μM of cisplatin (alone or in combination with the JNK inhibitor SP600125) upon addition of nutlin-3a. Thus, it can be stated that p53 is negatively associated with AKT activity, regardless of cisplatin concentration.

Next, to assess the involvement of AKT in p53 regulation, we analyzed the changes in p53 levels in cisplatin-treated cells affected by two different AKT inhibitors. In A549 cells exposed to 25 μM and 100 μM of cisplatin (both alone and in combination with the JNK inhibitor SP), addition of AKT inhibitor VIII (AKTi, 10 μM) or capivasertib (CAP, 10 μM) reduced p53 protein expression (Figure 12c–f). These results suggest that the AKT signaling pathway may positively mediate tumor suppressor p53 expression, as well as signaling in cisplatin-treated A549 cells.

Thus, a reciprocal interaction (negative feedback loop) was shown between the signaling molecules p53 and AKT, both in the absence and presence of JNK inhibition, regulating the fate of cisplatin-treated A549 cells.

## 3. Discussion

The reduction in drug dosage through drug combinations is essential to reduce drug toxicity while increasing treatment efficacy and potentially halting the development of drug resistance. Signaling molecules of mitogen-activated protein kinases (MAPK) and AKT kinase pathways mediate the cellular response to anticancer drugs and are candidates for improving the effectiveness of targeted and conventional chemotherapies [17].

Although the role of the MAPK family member JNK in regulating cancer cell viability has been widely studied and its potential role in modulating cell fate has been identified, JNK as a target in cancer therapy has not yet been used in the clinic [18]. In our work, the studies of combining inhibition of the protein kinase JNK with the DNA-damaging chemotherapeutic drug cisplatin were conducted with respect to the cisplatin concentration.

Before proceeding, we want to emphasize that non-small cell lung cancer A549 cells can be considered as intrinsically chemoresistant. Cisplatin concentrations used in this study may seem supraphysiologic and too high for clinical settings. Based on our findings, we named the cisplatin concentrations used in our work “low” and “high” (see Methods, Section 4.2). Under our conditions, for cisplatin-treated A549, it was 25 μM and 100 μM, respectively.

As published previously [6], JNK inhibition at low concentrations of cisplatin was very effective in killing non-small cell lung cancer A549 cells, and transcription factor p53 and survival kinase AKT have been shown to be involved in JNK signaling. Inhibition of JNK using different JNK inhibitors exhibited the same cisplatin dose-dependent effect on cell fate as demonstrated with SP600125, independently of the inhibitor used and regardless of their mechanism of action. Therefore, we can state that the inhibition of the JNK pathway determines the fate of the cell. In addition, JNK was found to be required for the proliferation of the cells studied. We would like to note that in A549 cells, the protective effect of JNK at low cisplatin concentrations persists, even after long-term in vitro passaging (50-100-200 passages; unpublished data). Thus, it is not determined by the genetic alterations in the A549 cells during cultivation in vitro.

Here, we aimed to address the potential mechanisms of the involvement of the protein kinase JNK in regulating cancer cell fate at different concentrations of cisplatin.

In present cytotoxicity studies, we evaluated the role of JNK in the A549 lung cancer cell line during the treatment with selected drugs—cisplatin, carboplatin, oxaliplatin, camptothecin, daunorubicin, doxorubicin, mitomycin C, and 5-fluorouracil—as described in the Methods. The rationale for the choice of these pharmaceuticals is provided in the Supplementary Materials S1, and notes on the selectivity of the inhibitors used in this work are delivered in Supplementary Materials S2. We exposed A549 cells to different drug concentrations with or without the JNK inhibitor SP600125. At a sublethal concentration of cisplatin, SP reduced the viability of A549 cells by approximately 70% (Figure 1a); however, at higher concentrations, the cell viability seemed to increase, suggesting the engagement of antiapoptotic/resistance mechanisms. Apoptotic mode of cell death, in addition to morphological evidence, was confirmed by the apoptotic cell death marker – PARP-1 cleavage (Supplementary Materials S3).

Cisplatin is primarily considered as an anticancer drug that forms different bifunctional covalent adducts within cellular DNA, with the highest preference for the N7 atom of guanine bases. Such adducts are recognized by mismatch repair proteins and other damage-recognition proteins. Nevertheless, the repair process often results in DNA double-strand breaks (DSBs), DNA damage signaling, and apoptosis [1]. Furthermore, upon cisplatin treatment, authors have reported elevated levels of autophagy in cisplatin-resistant cells compared to cisplatin-sensitive cancer cells [19]. Interestingly, in the mice model of acute kidney injury by cisplatin, autophagy inhibitors exacerbated cell death when a single high dose of cisplatin was administered, in contrast to low-dose treatment, suggesting different mechanisms of cellular damage induced by low versus high cisplatin doses [20]. Although we did not observe autophagy in our system, it is clear that there are certain differences between low and high cisplatin concentration-induced signaling in A549 cells.

In the case of carboplatin, when compared with other drugs used in this study, the viability assay results were most similar to those of cisplatin—at a sublethal concentration, SP reduced the viability of A549 cells very similarly (Figure 1b). There are data showing that JNK inhibition sensitized ovarian cancer stem cells to carboplatin [21], like in our experiments. On the other hand, in a mouse model, JNK activation was associated with better response to carboplatin [22]. JNK also potentiated cell death in carboplatin-treated lymphoma cells [23]. Different roles of JNK in oxaliplatin-treated cancer cells are known as well. Authors who investigated colon cancer HT29 cells concluded that JNK promoted autophagy and therefore was responsible for oxaliplatin resistance [24]. Alternatively, in colon cancer SW480 cells, it was demonstrated that oxaliplatin resistance was achieved through histone deacetylase SIRT1 suppressing the proapoptotic role of JNK [25]. In our experiments, SP gradually reduced oxaliplatin-treated A549 cell viability (Figure 1c), suggesting a protective role of JNK in A549 cells.

Due to systemic toxicity, TOP1 inhibitor camptothecin may be named as a poison, rather than a medication. It was shown in triple-negative breast cancer models that JNK may act proapoptotically in response to TOP1 poison treatment; however, some cells may remain indifferent [26]. In contrast, there are data that show that JNK favors the mitotic arrest of camptothecin-treated cancer cells to prevent cell death [27]. In our model, JNK inhibition did not affect the toxicity of camptothecin in the entire tested drug concentration range (Figure 1d).

There is a significant lack of data in the literature about daunorubicin-induced signaling. In this article, JNK inhibition significantly protected A549 cells at concentrations higher than LC50; however, there was no “SP effect” at low concentrations (Figure 1e). In line with our results, JNK was required for daunorubicin-induced apoptosis in acute myeloid leukemia models [28,29], as well as in healthy muscle stem cells [30]. In general, daunorubicin is known to generate reactive oxygen species to induce cell death through the activation of JNK [31].

In this study, in contrast to daunorubicin, its analog doxorubicin revealed an opposite—antiapoptotic—function of JNK (Figure 1f). In line with our results, SP had a sensitizing effect on several different cancer cell lines that were resistant to doxorubicin, both in vitro and in vivo [32]. In discordance with that, other authors found a proapoptotic function of JNK in doxorubicin-treated breast cancer cells [33]. However, such a discrepancy in the data may be at least partially explained by the different p53 status (normal or mutant) in different cancer cells [34]. As a reminder, A549 cells have an intact/wild-type *TP53* gene.

Data in the literature are very scarce regarding the connection between JNK’s role and mitomycin C-induced DNA damage. In A549 cell co-culture with T lymphocytes, it has been shown that mitomycin C increased PD-L1 expression in cancer cells and stimulated T cell anticancer immune activity. This process was somewhat dependent on JNK [35]. Our results of mitomycin C’s combination with SP were very similar to those of camptothecin, i.e., there were no significant changes in drug-induced lethality (Figure 1g).

Finally, our study showed that SP protected A549 cells from 5-FU toxicity in almost the whole range of concentrations used (Figure 1h). There are some reports about the JNK role in resistance to 5-FU in cancer. For example, in combination with some adjuvants, 5-FU could induce colon cancer cell death through the activation of the JNK/ROS pathway [36], and the JNK activator hesperidin sensitized CRC cells to 5-FU [37]. This is in line with our results.

The results of our experiments using the abovementioned chemotherapeutic drugs to investigate the JNK inhibitor “SP effect” in sensitizing cancer cells did not confirm the universal cell death induction mechanism originating from DNA damage. On the contrary, our research suggests that in combination cancer therapy, the combination partners for each drug can be different and specific to that drug. In the case of cisplatin, the results suggest that protein kinase JNK inhibition results in cell apoptosis only at a low cisplatin concentration. Thus, from a therapeutic perspective, JNK inhibition is compatible with cisplatin. However, distinct JNK signaling at a high cisplatin concentration contributes to cancer cell resistance. In this work, we aimed to clarify the network of key signaling events leading cancer cells to programmed cell death as a result of chemotherapeutic treatment.

Since we discovered the “SP effect” in *KRAS*-driven non-small cell lung cancer A549 cells, we hypothesized that KRAS might play a role in the protective effect of JNK against low concentration cisplatin toxicity. The authors already have suggested Ras as a JNK role switch in a drosophila model [38]. However, our experimental results using a novel, non-covalent, RAS-ON multi-selective, tricomplex inhibitor RMC-6236, also known as daraxonrasib, which targets mutant as well as wild-type variants of GTP-bound KRAS, NRAS, and HRAS [39], did not fully support this hypothesis. The work of Werner and colleagues, where wild-type homozygous *KRAS* gene restoration in A549 cells did not affect cell migration, ERK, and AKT activation, showcased that constitutive KRAS activation is not necessary for cells to stay tumorigenic—other deregulated pathways maintain the cancerous phenotype of the cells, regardless of whether the driver mutation can affect its own downstream effectors [40]. Studies confirm that factors such as mRNA splicing proteins and epithelial–mesenchymal transformation regulators could be more important in sustaining cancer cells than the heightened RAS activity itself [41,42]. Considering the A549 cell line as KRAS-independent [43] can explain why KRAS inhibition did not alleviate the “SP effect” in our experiments, as its activity may not be crucial for cell survival.

Results from JNK inhibition experiments in five other cancer cell lines differing in their tissue origins, mutation profiles, epigenomes, proteomes, metabolomes, and secretomes—colorectal adenocarcinoma DLD1 and HCT116 (both have a KRAS G13D mutation), small cell lung cancer SHP77 (KRAS G12V), chronic myeloid leukemia K562 (BCR-ABL1 fusion protein), and cutaneous squamous cell carcinoma A431 (EGFR-PPARGC1A fusion protein) [44,45]—showed varying degrees of cell viability reduction (Figure 3). Similar results were obtained by other researchers. Seino and colleagues detected JNK-mediated sensitization to cisplatin and paclitaxel in wild-type *RAS*-bearing ovarian cancer cells [46], while Lin and colleagues detected sensitization to cisplatin in three wild-type *RAS*-bearing (to our knowledge) hepatocellular carcinoma cell lines [47]. In contrast, eight primary lung cancer cell lines with untested *RAS* mutation status did not exhibit sensitization to cisplatin when treated with SP600125 [6]. Interestingly, in our experiments, A549, DLD1, and HCT116 cancer cell lines, all harboring a *KRAS* mutation, exhibited higher levels of sensitization to cisplatin compared with other tested cell lines. However, since KRAS inhibition did not abolish the “SP effect”, we cannot state that persistently activated KRAS is responsible for determining the “SP effect”.

To definitively claim the universality or conditionality of the “SP effect” phenomenon, additional experiments with a wide panel of wild-type *RAS*, as well as *KRAS*-, *NRAS*-, and *HRAS*-mutated cell lines, must be performed, including the introduction of different genetic *RAS* modifications.

In our model system, to confirm the involvement of cisplatin-induced DNA damage in determining cell fate, we used a histone variant H2AX (γH2AX) phosphorylation assessment by Western blot.

γH2AX phosphorylation and its magnitude indicate the presence and degree of DNA damage and are generally considered a marker of DSBs. In response to DNA damage, γH2AX is phosphorylated by phosphoinositide 3-kinase family kinases (PIKKs), ATM and ATM-Rad3-related (ATR) kinases, or by DNA-dependent protein kinases (DNA-PKcs). On the other hand, new data suggest a link between H2AX phosphorylation and oxidative stress. For instance, oxidative stress induced H2AX phosphorylation in human spermatozoa through the induction of DSBs [48]. Even more recent data show that, in addition to its role as a marker of DNA damage, H2AX is involved in the cellular fate decision between cell repair or death. It is known that phosphorylated H2AX recruits either proteins that are essential for the repair of damaged DNA or downstream signaling proteins involved in cell cycle and apoptosis regulation. Hence, this may be important for the initiation and execution of cell death [49].

Our studies have shown time- and dose-dependent H2AX phosphorylation and its inhibition by the ATM kinase inhibitor KU60019 in A549 cells treated with cisplatin (Figure 4). This confirms the role of ATM as an effector through which histone phosphorylation occurs. Our studies also confirm that ATM partakes in regulating cell viability in our cell model (Figure 5), but the molecular interactions that trigger apoptosis and whether H2AX is involved in them need to be further investigated.

Our next task was to assess the involvement of ATM in the cisplatin-induced JNK signaling pathway regulation in our model system.

Ataxia-telangiectasia mutated protein is the major kinase that phosphorylates H2AX in response to DNA double-strand breaks. Serine/threonine protein kinase ATM is known as the key regulator of cell survival and death following DNA damage. ATM activation involves autophosphorylation at Ser-1981 and the dissociation of inactive ATM dimers into active monomers. In addition to being a DNA damage sensor, ATM protein kinase is considered a central regulator of the DNA damage response (DDR). In response to DSBs, the Mre11/Rad50/Nbs1 complex recognizes the ends of DNA breaks and recruits ATM to the ends, where ATM self-activates through autophosphorylation. Then, ATM phosphorylates a number of proteins that are essential for regulating cell cycle checkpoints, activating DNA repair mechanisms, or inducing cell death, including BRCA1, CHK2, KAP1, AKT, and p53 [50,51]. Upon genotoxic damage, it is known that ATM acts as a switch from survival to apoptotic response, e.g., in chemoresistant cells [52].

However, in addition to DNA damage, ROS can be involved in ATM activation and activity. Cellular oxidative stress induced in response to DNA damage is a factor that modulates ATM signaling. Indeed, during oxidation, ATM switches to an active redox-sensing state, senses oxidative stress and organizes cellular antioxidant defense processes. Once oxidized, a specific residue of ATM cysteine-2991 is responsible for disulfide-linked dimerization, leading to autophosphorylation at serine-1981. In this case, ATM is activated in a non-canonical manner, involving intermolecular disulfide bonds in kinase dimers. Conformational changes in ATM upon oxidation have been identified. Also, studies show that ATM has a multifaceted role in maintaining redox balance: loss of ATM function leads to increased ROS, alterations in antioxidant processes, and impaired mitochondrial function in various cell types. The authors proposed that DNA damage induced reactive oxygen species’ production and activated ATM [53].

ATM inhibitor KU55933 was shown to increase ROS generation and to induce autophagy in cancer cells, and it played a cytoprotective role against ROS-mediated cytotoxicity. However, this ATM inhibitor reduced the viability of cisplatin-resistant head and neck cancer cells [54]. ATM-initiated autophagy in response to ROS protected HEK293 cells, while its inhibition with KU60019 was associated with increased endoplasmic reticulum stress. The latter data suggest a protective role for ATM in ER stress-mediated oxidative stress and mitochondrial apoptosis [55].

Recent studies have shown that a new pharmacological ATM inhibitor AZD1390 enhanced apoptotic effects in breast cancer cells at very low cisplatin concentrations (0.65–3.75 μM). Anti-proliferative effects were accompanied by increased intracellular ROS levels and compromised mitochondrial membrane potential. The studies using three cell lines demonstrated that AZD1390 affected ROS levels, depending on the inhibitor dosage administered [56]. When investigating the effects of ATM mutations on signaling pathways in response to cisplatin using CRISPR/Cas9, it was found that *ATM* knockdown enhanced cisplatin cytotoxicity by activating oxidative stress-induced senescence and necroptosis. A master regulator of the antioxidant response—NRF2—was downregulated by ATM inhibition, resulting in increased accumulation of ROS, and promoted cellular senescence [57]. Another study showed that ATM inhibitor KU55933 increased cellular ROS levels and protein oxidation, and also sensitized lung cells to TRX1 inhibitor auranofin. The authors highlighted the redox-active function of ATM in preventing ROS accumulation and the death of lung cells [58].

Thus, ATM is involved not only in the response to DNA damage but also in the sensing and modulation of oxidative stress.

On the other hand, data in the literature suggest that the level of DNA damage and the level of ATM activation are decisive factors in determining DNA damage-induced apoptosis. The extent of DNA damage may also determine different cell fates when ATM is inhibited. Thus, ATM is involved in protecting cells from death at low doses of chemotherapeutic drugs [56], and in the case of radiation [59,60].

As discussed above, the role of ATM may depend on reactive oxygen species. It was shown that ATM determined chemoresistance during mild chemotherapy with low ROS levels, and induced apoptosis at high-dose chemotherapy, possibly due to accumulation of ROS above a certain threshold [52].

Data in the literature reveal that in response to different genotoxic agents, activated ATM as well as ATR exhibit selective substrate specificity [61]. It is known that ATM is involved in the phosphorylation of more than 700 substrates in response to DNA damage, including p53 and AKT, among others.

p53 is a tumor-suppressing transcription factor involved in decision-making between different cell fates, including growth arrest, DNA repair, and apoptosis [60]. Whether it will be a p53-dependent DNA repair or cell death is usually decided by the level of DNA damage, just like in the case of ATM. Activated p53 promotes either survival signaling, cell cycle arrest in order to repair damaged DNA, or cell death by apoptosis during excessive DNA damage. Activation of p21 gene transcription occurs in the first case, while pro-apoptotic genes such as Bax, PUMA, FAS receptor, etc., occur in the second case. However, p53-independent DNA damage-induced apoptosis was observed in some cellular models [62].

The decision to induce a p53-dependent DNA repair or apoptosis is most probably dependent on the level of damage. For example, in the case of mild DNA damage by gamma irradiation, p53 enhanced base excision repair by directly interacting with the repair and recombination machinery, while severe damage suppressed the process [63].

The mechanism of cisplatin’s antitumor activity may also be related to its ability to alter the binding affinity of platinum-containing DNA to the active p53, as the DNA binding is essential for its tumor suppressor function. P53 has two DNA-binding sites, one of which (located at the C-terminus of the protein) binds to the damaged DNA. Cisplatin inhibits p53 binding to DNA at consensus and damaged sites. In addition, p53 is known to interact with cisplatin-modified DNA molecules [64,65,66,67,68].

The processes of direct and indirect p53 phosphorylation at serine-15, by which ATM can enhance its specific DNA binding, are potential mechanisms for the regulation of p53 activity. Recently, a novel mechanism involving ATM kinase has been identified, explaining the role of the p53 mRNA in the MDM2-dependent activation of p53 following DNA damage [69]. In addition, many other kinases follow phosphorylating p53 to stabilize, upregulate, and protect it from MDM2-dependent degradation [56,70]. Another mechanism by which p53 may control cell fate is proposed to be driven by controlled changes in p53 activation, like pulses of different amplitude following DNA damage [60].

As discussed above, ATM activation is a determining factor in DNA damage-triggered apoptosis. Depending on the extent of DNA damage and the level of ATM activation, the fate of a cell varies. Thus, just like ATM, depending on the extent of DNA damage, p53 can participate in determining different cell fates.

Typically, the extent of DNA damage determines the cell fate in a p53-dependent manner. The level of accumulated p53 in the cells may determine the initiation of a specific p53 pathway—low doses of DNA-damaging agents significantly increase p53-dependent base excision repair, while high doses inhibit it, leading to apoptosis. ATM was shown to regulate the stabilization and activation of p53, following DNA damage through the stimulation of both serine-15 phosphorylation and dephosphorylation of serine-376, as well as phosphorylation of MDM2, thus protecting p53 from MDM2-dependent degradation [71].

p53 activation by ATM is also modulated by ROS. Activation of the ATM-p53 signaling pathway was demonstrated in hepatocellular carcinoma cells during oxidative stress induced by naphthoquinone plumbagin [72]. Similarly, the pharmacologic inhibition of ATM activity resulted in increased mitochondrial activity and ROS levels that facilitated p53-dependent apoptosis [73]. It is clear from the existing data that multiple signaling pathways acting in parallel are involved in cell death.

Thus, both pro-survival and pro-death roles of ATM signaling in regulating cell fate have been described in the literature. In this work, we aimed to determine its role in tumor suppressor p53 and pro-survival protein kinase AKT regulation.

Our data indicate the same effect of ATM on p53 protein regulation at both low and high cisplatin concentrations (Figure 8). In our model system, the combination of low-concentration cisplatin with JNK inhibition induced an increase in p53 level over cisplatin alone; in both cases, p53 was inhibited by ATM inhibitor KU (Figure 8a), and this correlated with the slight increase in survival of A549 cells (Figure 5). In contrast, the combination of high concentration cisplatin with JNK inhibition reduced p53 levels compared to cisplatin alone, but again, the ATM inhibitor reduced p53 levels both when used with cisplatin alone and in combination with the JNK inhibitor SP (Figure 8b). Densitometric analysis of the Western blots presented in the article can be found in Supplementary Materials S4.

This indicates that ATM is required for p53 expression in cisplatin-treated cells in all cases studied.

The role of ATM in regulating protein kinase AKT has also been demonstrated in many cellular models. Evidence suggests that ATM regulates DNA damage-induced pro-survival signaling via AKT. For example, AKT was shown to be involved in transmitting the DSB-induced ATM-mediated signal to the MAPK protein kinase ERK. Authors suggested that at low DSB levels, a pro-survival signaling pathway involving ATM, AKT, and ERK was induced to promote DSB repair [74]. Similarly, in human colon cancer cells, ATM inhibition was shown to block AKT phosphorylation caused by auranofin [75].

Furthermore, ATM is known to control insulin-mediated signaling, which in turn regulates AKT signaling. It was suggested that glioma cancer cell growth and motility might be controlled by ATM via AKT, with ATM being an upstream mediator of radiation-induced AKT activation (phosphorylation of serine-473). ATM kinase inhibitor KU60019 radiosensitized glioma cells; compromised insulin, AKT, and ERK pro-survival signaling; and inhibited migration and invasion [76].

MAP kinases, including JNK, are also regulated in an ATM-dependent manner. Various data suggest that ATM acts upstream of JNK [77,78,79]. However, other studies have shown a dose-dependent effect: both low and high doses of cisplatin induced ATM-dependent phosphorylation of MKK4, a kinase responsible for JNK activation, but the low concentration did not induce JNK phosphorylation [52].

When examining AKT phosphorylation in our model, we found the same dependence on ATM as in the case of p53, although the changes in p53 and pAKT were opposite in the case of JNK inhibition, showing that JNK differently regulated p53 and AKT (Figure 9). Pharmacological ATM inhibition partially suppressed AKT phosphorylation in all cases studied. Thus, ATM was required for AKT phosphorylation at both low and high cisplatin concentrations, cisplatin alone or in combination with SP.

This suggests that the DNA damage signal transmitted by ATM is not responsible for the differential, cisplatin concentration-dependent involvement of JNK in regulating cell survival/death.

Another direction of our research was to investigate the role of ROS in our cellular model when combining cisplatin treatment with JNK inhibition. As mentioned earlier, in addition to nuclear DNA damage, the cytotoxic effect of cisplatin also includes the production of large amounts of reactive oxygen species in cells. Recent evidence indicates the importance of cisplatin-induced ROS. ROS are recognized as signaling molecules in many physiological processes. Low levels of ROS have often been shown in vitro to promote cell survival through the activation of mitogen-activated protein kinases (MAPKs); conversely, higher levels of ROS, induced by severe genotoxic stress, above a certain threshold cause a turnaround from resistance to apoptosis to stimulation of multiple death pathways, including apoptosis [80]. Increased levels of ROS disrupt cellular redox homeostasis; cause mitochondrial dysfunction, acting through peroxidation of mitochondrial membrane lipids, reduction in ATPase activity, disruption of ion distribution, etc.; and thereby contribute to cisplatin cytotoxicity.

Regarding cisplatin, it is known that cisplatin-treated cells accumulate excessive amounts of hydroxyl radicals and superoxide. ROS then activate various signaling pathways, including MAPK protein kinase JNK. It is documented that cisplatin-induced ROS generation further promoted DNA damage and thus contributed to the enhanced cytotoxic effect of cisplatin [9,81].

Data in the literature show the involvement of ROS in cisplatin signaling. Different ROS can be generated following different cisplatin concentrations, or depending on a cell type [82,83]. Cisplatin has been shown to induce apoptosis through both the death receptor and the intrinsic mitochondrial pathways in mouse proximal tubular cells and antioxidant N-acetylcysteine (NAC) was able to mitigate caspase-3 activation in this model [84]. Alternatively, activation of ROS-mediated survival signaling was reported in cisplatin-treated lung cancer cells [85,86].

Furthermore, an extremely high cisplatin concentration (1000 μM) was found to induce necrosis in tubular epithelial cells during cisplatin-induced acute nephrotoxicity. However, NAC was able to convert necrosis into apoptosis, a more favorable cell death variant [87].

NAC is a good candidate for the chemoprotection of cells, due to its ability to react with ROS [83]. However, in addition to its action as an antioxidant, the mechanisms of NAC’s cytoprotective effect include its high avidity for aqueous cisplatin species, preventing it from binding to other cellular targets. There are studies showing that NAC can bind to intracellular cisplatin and thereby reduce its reactivity [14]. Therefore, in our case, to demonstrate the role of ROS in cisplatin toxicity, we compared the results obtained with NAC with those obtained by treating cells with another antioxidant, DMTU, a scavenger of hydroxyl radicals. Hydroxyl radicals are among the reactive oxygen species induced by cisplatin [15]. Both antioxidants had similar effects on the cells we had tested (Figure 6b,c), confirming the involvement of ROS in cisplatin toxicity.

It is well known that ROS at different cisplatin concentrations can act as a signaling molecule or a cytotoxic agent. Depending on cisplatin concentration and cell type, cisplatin can generate different types of ROS and can also induce different cellular responses. Cisplatin cytotoxicity is positively correlated with an increase in ROS. At sub-toxic concentrations, cisplatin induces certain types of ROS, mainly superoxide and hydrogen peroxide, and to a smaller extent, hydroxyl radicals. Above a certain threshold, ROS are known to inhibit the cell cycle, leading to apoptosis and necrosis.

Understanding the molecular mechanisms of the cellular response to differential doses of cisplatin is crucial in making treatment decisions. Molecular studies show that tumor cells differently respond to cisplatin dosing, activating distinct transcriptional programs. For example, RNA sequencing followed by transcriptomic analysis revealed different enrichment of genes associated with specific pathways of cell functioning in colorectal cancer cells when comparing those treated with either a sub-toxic dose (30 μM) or acute dose (300 μM) of cisplatin. With low-dose treatment (but not high-dose), autophagy was activated, which served as a pro-survival strategy through the regulation of oxidative stress. As a result, the inhibition of autophagy increased the sensitivity to cisplatin [88].

Studies of the underlying mechanism of sub-toxic concentrations of cisplatin in regulating anoikis resistance indicated ROS generation (mainly superoxide and hydrogen peroxide) in H460 human lung carcinoma cells. Hydrogen peroxide induced by cisplatin exposure mediated the anoikis resistance in these cells [86].

P53 is known as a crucial factor activated in the process of cisplatin-induced cell-cycle arrest and apoptosis, both in cancer cells and in healthy tissues [13,84,89]: e.g., in renal cells, cisplatin-induced increase in ROS production contributed to p53 activation, leading to cell death. The authors proposed that cisplatin induced a rapid accumulation of hydroxyl radicals that led to the activation of protein kinases, which then phosphorylated and stabilized p53. ROS scavengers DMTU and NAC attenuated hydroxyl radical accumulation and diminished p53 activation in renal cells during cisplatin treatment. The results obtained suggested ROS involvement in p53 activation and tubular cell apoptosis [90]. The same antioxidants were proposed to be used for ameliorating the nephrotoxicity of cisplatin by other authors [15].

Considering various possible mechanisms, it is most likely that depending on the cisplatin concentration, ROS differentially promote oxidation of MDM2 and p53 protein cysteines, altering their affinity and interaction between them or with DNA, together with transformed MDM2-dependent p53 protein degradation, and at the same time, specific transcriptional activity of oxidized p53, as demonstrated by Hong et al. [91].

Our results showed that antioxidants augmented A549 cell viability during cisplatin treatment (Figure 6b,c). ATM inhibitor KU downregulated the p53 protein level in cisplatin-treated cells (Figure 8), confirming the role of ATM in p53 expression/stabilization, as discussed above. However, antioxidants either increased or decreased the p53 protein level, depending on the cisplatin concentration (Figure 10). Thus, both DNA damage signaling and ROS can regulate p53, either acting together or individually.

A complex relationship between ROS and AKT—another protein of interest in our research—signaling has recently been reviewed [80,92]. Studies have shown that AKT activation was mediated by ATM as a compensatory mechanism to cope with ROS [75]. ROS can be both inducers and by-products of autophagy [93,94]. In vivo, AKT signaling was demonstrated to control the amount of ROS during inflammation-induced autophagic destruction of bone tissue [95].

Our results showed that antioxidants either increased or decreased AKT phosphorylation in A549 cells, depending on the cisplatin concentration (Figure 11). However, the reduction in AKT phosphorylation (Figure 11b) did not result in reduced cell viability (Figure 6b,c). We will discuss this later; before that, let us overview some aspects of the stress kinase JNK.

c-Jun NH2-terminal protein kinase is characterized by diverse modes of its activation and action, and is also multifariously associated with ROS. JNK and ROS have a complex and bidirectional relationship in the cell. ROS are involved in upstream and downstream JNK signaling. The outcome of JNK action depends on the intensity and duration of its activation, interplay with other signaling pathways, and the cell/tissue type. Various aspects of possible JNK involvement in regulating cell death; its inhibition pathways and mechanisms, efficacy, and the side effects; and approaches of developing JNK-targeting strategies in a clinical perspective were discussed and presented in our previous article [6].

The stress kinase JNK is known to regulate oxidative stress; the inhibition of JNK results in increased ROS production [96]. JNK can modulate ROS levels by regulating the expression and activity of antioxidant enzymes, such as catalase and superoxide dismutase. On the other hand, generated ROS are potent activators of JNK by inducing oxidative modifications of its upstream components, the kinases MAPKK and MAPKKK [97,98]. The JNK2 isoform is suggested to be antiapoptotic, while JNK1, in some cases, can be proapoptotic [99]. Furthermore, JNK is known to be responsible for cancer cell resistance to cisplatin, e.g., in ovarian cancer [46].

Our current research has shown that JNK may be involved in the regulation of ROS in cisplatin-treated A549 cells. Our findings are consistent with the data in the literature, where active JNK is shown to downregulate ROS levels in cells. For example, by using Rat-1 fibroblasts as a model, it has been shown that JNK1, being involved in diverse cellular functioning, was implicated as a cell sensor of redox stress. JNK1 participated in stress response via inhibition of cellular H_2_O_2_ production. Authors suggested that JNK1 was involved in ROS regulation through JNK1-directed gene expression or by direct effects on ROS-generating systems and enzymes involved in ROS metabolism, such as NADPH oxidase, superoxide dismutase, and glutathione peroxidase. JNK1 could also affect the cellular response to H_2_O_2_ by regulating the expression and activity of antioxidant enzymes. The pharmacological JNK inhibitor SP600125 reversed this action [100].

Other works showed that SP600125 promoted ROS generation and therefore increased cell death by necroptosis in non-small cell lung cancer (NSCLC) cells. In H_2_O_2_-treated Calu-6 and A549 cells, among the MAPK inhibitors, only the JNK inhibitor significantly augmented the loss of mitochondrial membrane potential and increased the ROS levels during the first hour of treatment [101]. However, SP600125 was shown to reduce ROS production in four different glioma/neuronal cell line models, and it protected the cells from oxidative stress by inhibiting JNK or its downstream targets [102,103].

Therefore, the effect of SP600125 on ROS is complex and variable, and it may depend on factors such as the dose, duration, and mode of administration of SP600125; the source and type of ROS; the antioxidant capacity of the cells; and the interaction with other signaling pathways.

c-JUN and P53 transcription factors are among the proapoptotic targets of activated JNK. Our earlier results demonstrated different roles of JNK in p53 activation, depending on the cisplatin concentration in A549 cells [6].

The role of JNK in the transcription factor p53 activation has been investigated by using various DNA-damaging agents, including chemotherapeutic drugs. Differential JNK-mediated phosphorylations of p53, which occurred in a DNA damage-dependent manner, were reported in different cells. They were responsible for stabilizing the p53 protein and enhancing its transcriptional activity, including threonine-81, serine-34, and serine-15 [78,104]. JNK-specific phosphorylation of p53 at serine-6 is also known to occur in response to ionizing radiation or etoposide exposure [105], as well as in formyltetrahydrofolate dehydrogenase-mediated apoptosis in A549 cells [106]. In our case, p53 at serine-6 phosphorylation correlated with the amount of total p53 protein [6]. Regarding the participation of ROS, there are data that show that ROS production promoted JNK activation and facilitated p53 function upon its release from MDM2. As a result, activated p53-induced prooxidant genes increased ROS levels, further activating JNK and thereby enhancing p53 activity [98].

Two different mechanisms of the JNK-mediated regulation of p53 levels have been described. Direct JNK binding to p53 has been shown to contribute to p53 ubiquitin-mediated degradation, while JNK-mediated phosphorylation of p53 led to its accumulation and activation [107,108].

In our studies, depending on the cisplatin concentration, the inhibition of JNK had a differential effect on AKT phosphorylation as well. It is known that AKT and JNK pathways regulate many biological processes in concert, such as proliferation, migration, and apoptosis, and are important in cancer development [109]. Both positive and negative feedback between them is documented [110,111,112,113].

While evaluating the role of ROS in regulating the tumor suppressor p53 and the pro-survival kinase AKT in our cell model system in the presence of low or high cisplatin concentrations, we have found that ROS scavengers decreased the p53 protein level at low cisplatin concentrations in A549 cells, but increased it at high concentrations (Figure 10). Two different ROS inhibitors, NAC and DMTU, reduced p53 levels in cells treated with a low cisplatin dose, with and without the JNK inhibitor. Conversely, at a high dose of cisplatin, antioxidants increased the levels of p53 both with and without the JNK inhibitor. Thus, depending on the cisplatin concentration, ROS were differently involved in p53 regulation. As JNK inhibition increased ROS levels, using both cisplatin concentrations in our tested cells (Figure 6a), it seems that p53 is inversely regulated by JNK and ROS. Supposedly, the targets of these signaling molecules in the regulation of p53 may differ and change with increasing cisplatin concentrations.

The same can be said about the regulation of AKT depending on JNK, as well as AKT dependency on ROS. ROS scavengers promoted AKT kinase phosphorylation at low cisplatin concentrations alone or in combination with the JNK inhibitor SP600125, but suppressed AKT phosphorylation at high concentration (Figure 11). Accordingly, in this case, the targets of JNK and ROS in the regulation of AKT differed and changed with changing cisplatin concentrations. Another possibility is that p53 and AKT are in the same signaling pathway regulating cell death and are interdependent. Our data confirms the interrelation between p53 and AKT.

One possible explanation for the different dose-dependent role of JNK in regulating cell death would be the different nature of JNK interaction with p53, an apoptosis-regulating protein, at different cisplatin concentrations. As mentioned above, direct JNK binding to p53 has been shown to contribute to p53 ubiquitin-mediated degradation, while the JNK-mediated phosphorylation of p53 leads to its accumulation and activation [107,108].

JNK-dependent ROS differentially regulate p53 at low versus high doses of cisplatin combined with the JNK inhibitor. In other words, there is a different role of ROS in p53 activation at low and high cisplatin concentrations. We think that ROS is a threshold regulator that, together with an as-yet-unidentified factor, can differentially regulate p53, which may be a switch and an executor of cell death. We believe that the differentially activated p53 may be a molecular switch in the regulation of the JNK role, from antiapoptotic to neutral or proapoptotic.

Thus, based on these data, it can be assumed that in cells without cisplatin or at low cisplatin concentrations, JNK inhibition increases the amount and/or activity of p53 protein, which in turn inhibits the activity of the AKT survival signaling pathway. On the other hand, at high concentrations of cisplatin, JNK is involved in phosphorylating p53, thereby stabilizing and activating it. Therefore, the inhibition of JNK stops this process, reducing the amount of p53 protein. As a result, AKT phosphorylation is restored, and cells become resistant to cisplatin. Taken together, our data suggest that the differential role of the JNK signaling pathway in modulating cisplatin-induced cytotoxicity is likely due to its ROS-dependent regulation of p53 functions. Our data on opposing changes in AKT and p53 activation in regulating the sensitivity of A549 cells to cisplatin in combination with JNK inhibition raised questions about the possible interaction between these molecules.

By studying the relationship between the tumor suppressor p53 and the pro-survival protein kinase AKT in cisplatin-treated A549 cells, reciprocal interaction between these signaling molecules was identified in both the absence and presence of JNK inhibitor SP.

There are different interplays of p53 and AKT described in the literature. Firstly, in response to stress, p53 is known to initiate the transcription of many response genes, including tumor suppressor PTEN, which is a phosphatase of AKT [114]. For example, p53 activator nutlin-3 sensitized lung cancer cells to axitinib by repressing the AKT1/WNT signaling axis [115]. These are examples of p53-dependent AKT inhibition. Secondly, active AKT can phosphorylate MDM2, which then localizes to the nucleus and targets p53 for degradation [116], resulting in AKT-dependent p53 inhibition. Both mechanisms present a negative p53-AKT feedback loop. However, failed clinical trials, where PI3K-AKT pathway inhibitors were tested, revealed that PI3K, AKT, and mTOR were required for p53-mediated cancer cell death [117]. In 2010, another research group reported what may have been the first ever observation that the widely used chemotherapeutic medication adriamycin (doxorubicin) induced DNA-damage-extent-dependent signaling, which was mediated by the PI3K-AKT-mTOR pathway. They demonstrated that the AKT signaling inhibitor LY294002 prevented p53-regulated proapoptotic gene transcription upregulation at low concentrations of doxorubicin, leading the cells to survive; however, LY294002 had no such effect at high doxorubicin concentrations [118].

A non-canonical positive feedback loop mechanism has been recently explained. The authors have demonstrated that genotoxic stress activated AKT in the cell nucleus through a p53-dependent mechanism where p53, AKT, FOXO3, and phosphoinositides formed the complex termed “p53-PI signalosome” at DNA damage sites, together with H2AX [119].

In our case, the activation of p53 by nutlin-3a resulted in AKT inhibition (Figure 12a,b), while AKT inhibition resulted in p53 inhibition (Figure 12c–f), regardless of JNK inhibition and cisplatin concentration. This finding reveals another unconventional example of the interaction between p53 and AKT.

These data allow us to propose a hypothesis about the dependence of JNK’s role in cell functioning on the cisplatin concentration, based on the observation of decreased p53 levels and an increased AKT phosphorylation at high cisplatin concentrations. Ergo, the loss of p53 function can lead to AKT activation. Our results indicate that p53 inhibition must precede AKT activation to corrupt chemotherapy sensitivity at high cisplatin concentrations. This may be due to ROS, which cause p53 downregulation at high cisplatin concentrations. Consequently, cancer cell sensitization is lost.

Thus, our work has provided more clarity on the dependence of the role of the MAP kinase JNK on the concentration of cisplatin. The differential JNK-dependent ROS impact on p53 functions appears to be responsible for changes in AKT phosphorylation and, in turn, DNA damage-induced cytotoxicity. However, the precise molecular mechanism responsible for the change in the role of JNK from antiapoptotic to neutral/proapoptotic depending on the concentration of chemotherapeutic drugs has not yet been definitively determined.

## 4. Materials and Methods

### 4.1. Chemicals

Cisplatin (#479306), carboplatin (#1096407), camptothecin (#C9911), mitomycin C (#M0440), resazurin (#R7017), MTT (#M2128), N-acetylcysteine (2 mM, #A9165), N,N′-dimethylthiourea (1 mM; #D188700), H2DCFDA (100 μM, #D6883), and nutlin-3a (10 μM; #SML0580) were from Sigma-Aldrich, St. Louis, MO, USA. Other reagents: 99.5% dimethylsulphoxide DMSO (#7029.1) from Carl Roth, Karlsruhe, Germany; oxaliplatin (5 mg/mL infusion solution) from Fresenius Kabi Oncology Plc., Hampshire, UK; daunorubicin (#251800) and AKT inhibitor VIII (10 μM, #124018) from EMD Millipore/Merck, Billerica, MA, USA; ATM inhibitor KU60019 (5 μM, #S1570) and RAS inhibitor RMC-6236 (1 μM, #E1597) from Selleck Chemicals LLC, Houston, TX, USA; SP600125 (20 μM; #J67272) from Alpha Aesar, Ward Hill, MA, USA; capivasertib AZD5363 (10 μM; #15406) from Cayman Chemical, Ann Arbor, MI, USA.

### 4.2. Cell Culture

The human cell lines—lung adenocarcinoma A549 (Cell line Service GMBH, Eppelheim, Germany, #300114; male), chronic myeloleukemia K-562 (ATCC, Manassas, VA, USA, #CCL-243 TM; female), small cell lung carcinoma SHP-77 (ATCC #CRL-2195 TM; male), skin epidermoid carcinoma A431 (female; a gift from the Department of Bioelectrochemistry and Biospectroscopy, Life Sciences Center, Vilnius University), and colon adenocarcinoma DLD-1 and HCT116 (both male; a gift from the Proteomics Center, Vilnius University)—were cultured in Iscove’s modified Dulbeco’s medium (IMDM; Corning Mediatech, Manassas, VA, USA, #10-016-CV), supplemented with 10% heat-inactivated fetal bovine serum (Gibco, Paisley, UK #10500064) and 1% antibiotic–antimycotic (Corning #30-004-CI) in 5% CO_2_ 37 °C incubator. Adherent cells were split by the application of trypsin/EDTA solution (Corning #25-053-CI) and suspension cells were split by diluting them with the cell culture medium. Cell washing was done with phosphate-buffered saline (1xPBS) (Corning, #20-031-CV).

The main tests were performed using the chemotherapy drug cisplatin. Based on our findings, we named the cisplatin concentrations used in our work as “low” and “high”. The sublethal (subthreshold) concentration of cisplatin (“low”) was the concentration at which JNK exhibits a strong antiapoptotic effect. When the concentration was increased 2–4-fold (“high”), the role of JNK changed to a proapoptotic one. At the threshold, the high cell death activity, induced by JNK inhibition, changed to a neutral/reverse response, and the activation of the signaling molecules p53 and AKT changed to the opposite.

### 4.3. Cell Viability Assay (MTT Method)

The method is based on the reduction of yellow soluble 3-(4,5-dimethylthiazol-2-yl)-2,5-diphenyltetrazolium bromide (MTT) into insoluble purple formazan crystals, which is carried out by cellular dehydrogenases. The method was used only to assess the viability of adherent cells. The cells were counted using a Neubauer-improved counting chamber (Paul Marienfeld GmbH & Co.KG, Lauda-Königshofen, Germany) and seeded at a density of 25,000 cells/well in 96-well plates. Then, 24 h after seeding, the medium was aspirated from the control samples, and 100 μL of 0.2 mg/mL MTT (Sigma–Aldrich) dissolved in PBS was added. After 1.5 h in the incubator, the MTT was aspirated, and the wells were left empty until measurement, together with other wells after the intended treatments. The formed formazan remained inside the adhered cells. The medium was replaced in the other wells, and the exposures of selected drugs and inhibitors were performed. After 72 h incubation in the CO_2_ incubator, the medium was replaced with 100 μL of 0.2 mg/mL MTT, just like for the control samples. To dissolve the crystals, 100 μL of ethanol was added to each well. Absorbance at 570 nm was measured by using a plate reader, Varioskan Flash from Thermo Fisher Scientific Oy, Vantaa, Finland.

“Relative cell viability” in the figures is cell viability after 72 h treatment normalized by initial viability, in relative units. In addition, 1.0 means the viability in 72 h is the same as at the beginning of the experiment (0 h).

### 4.4. Cell Viability Assay (Resazurin Method)

The method is based on the reductive reaction of weakly fluorescent resazurin to fluorescent resorufin, which takes place in the mitochondria of living cells. The method was used to assess the viability of K-562 suspension cells and A549- and HCT116-adherent cells. The cells were counted using a Neubauer-improved counting chamber and seeded at a density of 25,000 cells/well in 96-well plates. After either 1 h (in the case of K-562 cells) or 24 h (in the case of A549 and HCT116 cells), the fluorescence of the zero control wells was measured with the dye. Other wells (without the dye) were treated with the drugs and inhibitors and incubated for 72 h in the CO_2_ incubator. In each well containing 100 μL of medium, 5 μL of 1 mg/mL reagent was added, gently mixed by pipetting to ensure even distribution of the dye, and incubated for 1 h in a thermostat. Resorufin fluorescence (excitation 540 nm, emission 590 nm) was measured using a Varioskan Flash plate reader (Thermo Fisher Scientific Oy, Vantaa, Finland).

### 4.5. H2DCFDA Fluorescence Measurement

The cells were seeded into a 96 well plate. The next day, the medium was replaced, and the cells were treated with cisplatin and SP600125. Then, 20 h later, H2DCFDA (in DMSO) was added to the same wells to make 100 μM of its final concentration. As a control, half of the plate was incubated with DMSO only. After incubating cells with fluoresceine for 2 h, the medium was removed and the wells were washed twice with PBS, performing the fluorescence measurement in fresh PBS. The control wells (without H2DCFDA) served as blanks for each group of treatment. Excitation 490 nm, emission 545 nm, measurement 200 ms.

### 4.6. Protein Extraction

For protein analysis, 24 h after treatment, only adherent cells were lysed in lysis buffer (10 mM TrisHCl buffer (pH 7.4) containing 50 mM NaCl, 5 mM EDTA, 50 mM NaF, 1% Triton X-100, 1 mM PMSF, 2 mM Na3VO4 and 20 μg/mL aprotinin). The protein concentration was equalized within the experimental group, using the Bradford assay measuring 595 nm absorption in the plate reader. The final samples were denatured by adding SDS and β-mercaptoethanol and heating for 5 min. at 95 °C.

### 4.7. Western Blot Method

Equal amounts of protein were resolved in SDS-PAGE (sodium dodecylsulphate polyacrylamide gel electrophoresis in Tris-glycine buffer). The proteins were transferred onto a PVDF membrane using a semi-dry system Power Blotter (Invitrogen). Phosphorylated signaling molecules were visualized by using antibodies and an enhanced chemiluminescence detection system (BioRad Clarity substrate and horseradish peroxidase (HRP)-conjugated antibodies, Carestream Health X-ray blue film). Representative Western blots from at least 3 independent experiments which resulted in a similar outcome are presented in this article. The total protein staining in PAA gel with Coomassie brilliant blue dyes (Imperial TM Protein Stain and/or PageBlue Protein Staining Solution; Thermo Scientific, Rockford, IL, USA) served as loading controls. The horseradish peroxidase-conjugated secondary antibodies (goat anti-rabbit and goat anti-mouse; 1:5000) were obtained from BioRad Laboratories, Inc. (Hercules, CA, USA). Primary antibodies against phospho-AKT (Thr308) (#2965, 1:1000) were from Cell Signaling Technology (Beverly, MA, USA), phospho-gamma-H2AX (Ser139) (#26350, 1:2000) were from Abcam (Cambridge, UK), and p53 (#sc-6243, 1:300) was from Santa Cruz Biotechnology (Dallas, TX, USA).

### 4.8. Statistical Analysis

Statistical calculations and graph visualizations were generated using RStudio (2026.01.1+403 "Apple Blossom" (R version 4.5.0)). Quantitative data were obtained from at least three independent biological experiments with at least four technical replicates. The results were evaluated by performing two-way ANOVA analysis with Tukey’s HSD post hoc correction (residuals vs. fitted plot, Levene’s test, and the Fligner–Killeen test were used to assess data variance. Q-Q plot and Shapiro–Wilk tests were performed to assess data normality; tidyverse (v. 2.0.0), car (v. 3.1-5), and qqplotr (v. 0.0.7) packages were used). In the case of H2DCFDA fluorescence, the graph presented is from one experiment that is representative of at least ten independent experiments performed in at least four technical replicates. All graphs visualize the means, with error bars representing the standard deviation (SD). The data were normalized by equating the cell viability of untreated controls to 1. Differences were considered statistically significant at *p* < 0.05 (*), *p* < 0.005 (**), *p* < 0.0005 (***).

Note: In some graphs, statistical significance is indicated with “0” symbols. Because ANOVA is sensitive to violations of the homogeneity of variances assumption, the initial analysis using the complete dataset (including the 0 μM concentration groups) did not reveal the significance for certain comparisons. The 0 μM concentration groups exhibited substantially greater variability and were therefore excluded in a secondary analysis to evaluate whether this variance affected the outcome. Newly detected significances are marked with “0” symbols corresponding to *p* < 0.05 (0), *p* < 0.005 (00), and *p* < 0.0005 (000). To clearly distinguish these results from those derived from the full dataset, which are marked with “*”, we used separate symbols. These secondary calculations were performed to better characterize the data tendencies and aid interpretation, while acknowledging the limitations of the small sample size and heterogeneity of variance.

The biological replicate data are shown in Table 1.

**Table 1:** The biological and technical replicate data, presented in the figures and used for statistical analysis.

Figure	Condition	N	Replicates in Each Experiment
Figure 1a	Cisplatin	5	4
Figure 1b	Carboplatin	3	4
Figure 1c	Oxaliplatin	3	4
Figure 1d	Camptothecin	3	4
Figure 1e	Daunorubicin	4	4
Figure 1f	Doxorubicin	3	4
Figure 1g	Mitomycin C	3	4
Figure 1h	5-Fluorouracil	4	4
Figure 2	RMC-6236	4	4
Figure 3a	A549	7	4
Figure 3b	DLD-1	3	4
Figure 3c	HCT116	3	4
Figure 3d	SHP77	3	4
Figure 3e	K562	4	4
Figure 3f	A431	4	4
Figure 5	KU6	3	4
Figure 6a	H2DCFDA **	1	6
Figure 6b	NAC	3	4
Figure 6c	DMTU	4	4

** H2DCFDA experiment is the only exception, when Student’s *t*-test was used instead of ANOVA.

## 5. Conclusions

Our previous combination therapy studies showed the change in the role of the MAP kinase JNK’s signaling in modulating chemotherapeutic drug cisplatin-induced cytotoxicity and the regulation of signaling proteins, pro-death transcription factor p53 and pro-survival kinase AKT, in lung cancer A549 cells in a cisplatin concentration-dependent manner. There, we aimed to determine the prevalence of our identified phenomenon in cisplatin-treated lung cancer cells.

Current research involved a broad spectrum of anticancer drugs that cause DNA damage (platinum drugs, topoisomerase inhibitors, mitomycin C, 5-FU, etc.). Here, we demonstrated that the DNA damaging drugs did not share a common mechanism that would lead to a switch in the role of JNK from antiapoptotic to neutral or proapoptotic. This property was characteristic to cisplatin, a platinum-containing compound.

Using small molecule inhibitors of RAS, ATM, AKT, and MDM2, as well as antioxidants NAC and DMTU, we extended the scope of our previous study to investigate the causes and potential molecular mechanisms responsible for the switch in the role of JNK from antiapoptotic to neutral/proapoptotic, depending on the concentration of cisplatin, the DNA-damaging drug. This dependence of the role of JNK on the drug concentration is a new and yet underexplored phenomenon. Importantly, the effect was not unique to A549 cells, as it was observed in other types of cancer (lung, skin, colon) cell lines with mutated KRAS, but it was not universal for all cancer cell lines tested.

Previously, AKT and p53 were suggested to be the signaling proteins mediating the outcome of JNK inhibition at different cisplatin concentrations in A549 cells. The current research showed that p53 negatively regulated AKT phosphorylation, and AKT signaling positively mediated tumor suppressor p53 expression, showing a novel, non-canonical reciprocal interaction of p53 and AKT in A549 cells. DNA damage and generated reactive oxygen species were identified to be differently involved in the regulation of p53 and AKT activation.

Inhibition of DNA-damage-sensing protein kinase ATM decreased p53 expression and AKT phosphorylation in the same manner at both low and high cisplatin concentrations, with cisplatin alone or in combination with JNK inhibitor SP600125, indicating that DNA damage signaling through ATM should not be responsible for the differential, cisplatin concentration-dependent involvement of JNK in regulating cell survival.

The protective effect of the antioxidants on the viability of A549 cells exposed to low concentrations of cisplatin showed that ROS were involved in cytotoxic processes at sublethal cisplatin concentrations, both in the presence and absence of JNK inhibitor SP. At different cisplatin concentrations, ROS were differently involved in regulating the activation of cell death/survival signaling molecules p53 and AKT, by doing it in opposite directions. Accordingly, ROS scavengers decreased the total p53 levels but promoted AKT phosphorylation at low cisplatin concentrations, alone or in combination with the JNK inhibitor SP; and vice versa – these antioxidants increased the level of p53 but suppressed AKT activation at a high cisplatin concentration.

Taken together, our data suggest that the drug concentration-dependent differential role of the JNK signaling pathway in modulating cisplatin-induced cytotoxicity is manifested through the regulation of p53 functions, which is ROS-dependent. As a result, AKT phosphorylation is altered. The differentially activated p53 in response to ROS at low versus high concentrations of cisplatin, combined with SP600125, may be a molecular switch in the role of JNK from antiapoptotic to neutral/proapoptotic. Our data encourage a further, more thorough investigation of this phenomenon of drug concentration-dependent changes in the role of JNK, as well as the search for potential mediators and switches that are responsible for p53 regulation in cancer cell chemoresistance.

## Data Availability

The original contributions presented in this study are included in the article/Supplementary Material. Further inquiries can be directed to the corresponding author.

## Supplementary Materials

The following supporting information can be downloaded at: https://www.mdpi.com/article/10.3390/ph19030509/s1. Original Blots and Gels.pdf—uncropped images of blots and gels; Supplement.pdf—DNA-damaging drugs (Supplementary Materials S1), Inhibitor specificity (Supplementary Materials S2), Apoptotic markers (Supplementary Materials S3), and Densitometry of Western blots (Supplementary Materials S4) [120,121,122,123,124,125,126,127,128,129,130,131,132,133,134,135].

## Abbreviations

The following abbreviations are used in this manuscript:

5-FU	5-fluorouracil, synthetic antimetabolite
AKT	Protein kinase which mostly transduces survival signaling
ATM	DNA-damage-sensing protein kinase, “ataxia-telangiectasia-mutated”
CAP	Capivasertib, inhibitor of protein kinase AKT
DDR	DNA damage response
DMTU	N,N′-dimethylthiourea, ROS scavenger
DSBs	Double-strand breaks
H2AX	Histone 2A variant, the sensor of double-strand DNA breaks
JNK	Jun-N terminal Kinase, stress-activated protein kinase of MAPK family
KU	Inhibitor of ATM, KU60019
MAPK	Mitogen-activated protein (MAP) kinase
NAC	N-acetyl cysteine, synthetic antioxidant
NUT	Nutlin-3a, inhibitor of MDM2, thus activator of p53
ROS	Reactive oxygen species
SP	Synthetic compound SP600125, Anthra [1,9-cd]pyrazol-6(2H)-one; JNK inhibitor
“SP effect”	The phenomenon of the efficient sensitization of cancer cells to only low chemotherapeutic drug concentrations using SP600125, along with the reversal of its effect to neutral or the opposite at high drug concentrations
