# Supplementary material for: Potential Mechanisms of MAP Kinase JNK’s Involvement in Modulating Cancer Cell Fate in a Cisplatin Concentration-Dependent Manner
Source: Pharmaceuticals (Basel). 2026 Mar 20;19(3):509. doi: 10.3390/ph19030509 (PMC13028654; doi:10.3390/ph19030509)

Supplementary Materials: S1, S2, S3, S4

DNA-damaging drugs

Supplementary Materials S1

To explain the rationale of using various chemotherapeutic drugs in our screening experiments, we want to highlight their main features and the known roles of JNK in cancer treatment.

Cisplatin is one of the oldest chemotherapeutic drugs in the world (approved in 1978) currently used for breast, ovarian, testicular, head and neck, esophageal, lung, bladder, and brain tumors. This platinum compound has a strong anticancer effect, but at the same time it also damages healthy cells in the body resulting in severe kidney and brain damage, and hearing loss. At the same time, cisplatin is accompanied by intrinsic, inherent, and acquired resistance in tumor cells, limiting its effectiveness [1]. Due to its high reactivity, most cisplatin molecules bind to blood serum proteins and lose their therapeutic effect, and only a small amount of cisplatin reaches cancer cells. This is the reason why low dosage of cisplatin is not effective in cancer patients. Transcription inhibition caused by DNA damage in the nucleus is considered to be the main mechanism of action of cisplatin. Additionally, damage to mitochondrial DNA also disrupts the expression of electron transport chain genes, causing oxidative stress and subsequent damage to proteins, lipids, and nucleic acids [7] [8]. Although it is known that the action of cisplatin usually occurs through the activation of signaling pathways, which are cell type specific, the data obtained during global proteome and phosphoproteome analysis of A549 cells indicated possible cisplatin-induced direct damage to protein kinases and phosphatases [120].

Cisplatin-treated cells accumulate excessive reactive oxygen species (hydroxyl radicals and superoxide), ROS then activate JNK. JNK2 is suggested as a potential cause of cisplatin resistance, while JNK1 in some cases can be proapoptotic [100]. JNK is known to be responsible for cancer cell resistance to cisplatin, e.g. in ovarian cancer [46]. Upon cisplatin treatment, in some cases, autophagy induction has been detected, in cisplatin-resistant cells being more profound when compared to cisplatin-sensitive cancer cells [19]. Interestingly, in mice model of acute kidney injury by cisplatin, autophagy inhibitors exacerbated cell death when a single high dose of cisplatin was administered, in contrast to low dose treatment, suggesting different mechanisms of cellular damage induced by low versus high cisplatin doses [20].

On the other hand, the influence of cisplatin interaction with the cell plasma membrane cannot be ruled out. It is known that cisplatin interacts with membrane lipids and induces transient lipid phase shifts and membrane fluidity changes. Given that cell signaling events depend on the biophysical properties of the membrane, changes in its organization and structure caused by platinum and lipid membrane interaction may affect cell signaling events leading to cancer cell death [121], [122].

Carboplatin and oxaliplatin are second- and third-generation platinum-based chemotherapeutic agents, respectively. One of the main differences between cisplatin and other platinum drugs is the type and amount of DNA adducts they form. DNA adducts are the covalent bonds between platinum and DNA bases, which can cause DNA damage and trigger cell death. Due to the more stable structure of carboplatin, water replaces the leaving group in the cell more slowly. It also forms less dangerous DNA crosslinks. Oxaliplatin contains a lipophilic group, which allows it to enter the cell more efficiently by passive diffusion. As in the case of carboplatin, the leaving group of oxaliplatin is replaced by water in the cell much more slowly than the chloride ligands of cisplatin. Due to the same diaminocyclohexane group, oxaliplatin forms additional hydrogen bonds to the DNA binding, which strongly bend the DNA chain. Such bending is not present when cisplatin and carboplatin are used. Due to their slower drug activation and lower toxicity, these two drugs cause

fewer side effects than cisplatin [124]. Cisplatin forms mainly intrastrand crosslinks between adjacent guanines, while oxaliplatin forms mainly interstrand crosslinks between opposite guanines. Although oxaliplatin is approved only for colorectal cancer (in combination with 5-FU), due to the different mechanisms of entry into the cell and different DNA damage, it can be used to treat tumors resistant to cisplatin or carboplatin. Carboplatin and oxaliplatin are better tolerated with less nephrotoxicity and ototoxicity [125]. Interestingly, oxaliplatin also forms some monofunctional adducts that do not crosslink DNA strands, and oxaliplatin adducts are more stable and less repairable [126].

Camptothecin is a natural anticancer compound which primarily inhibits topoisomerase 1 (TOP1). Its planar 5-ring structure intercalates into DNA and prevents the enzyme from cutting the single strand of DNA. Due to low solubility, inactivation by hydrolysis and systemic toxicity, only its derivatives (topotecan, irinotecan) have been approved to be used in clinic [127]. JNK involvement in ToOP1 poison management is well established [26] [128].

Daunorubicin and doxorubicin (Adriamycin) are anthracycline analogs. They are classified as topoisomerase 2 (TOP2) inhibitors and induce accumulation of irreparable DNA breaks. Daunorubicin is used only to treat acute leukemias. Due to the specific molecular structure, intercalated daunorubicin is known to bend DNA, causing histones, including H2AX, to dissociate. Because of that, DNA damage signaling through H2AX phosphorylation by protein kinase ATM is delayed. Alternatively, anthracyclines can disrupt oxidative balance by interacting with the electron transfer chain proteins inside the cells, causing accumulation of ROS [129] [130]. There is some data available about JNK involvement in doxorubicin-induced A549 cell apoptosis [131].

Mitomycin C, like daunorubicin, was extracted from bacteria of the genus *Streptomyces* and belongs to a large class of antibiotics called mitomycins; it is also a chemotherapeutic drug. Due to bone marrow suppression, cardiotoxicity, and other side effects, mitomycin C is usually used at low concentrations in combination with other chemotherapeutic agents. Mitomycin C is a prodrug that, once activated, forms DNA adducts. It also inhibits thioredoxin reductase (TRXR) and increases oxidative stress [133]. Interestingly, the synthetic lethal effect of the low dose of p53 reactivating molecule RITA in combination with TRXR inhibition was mediated by JNK, and apoptosis was prevented by JNK inhibitor [99]. Furthermore, Mitomycin C was shown to induce JNK-dependent cell death receptor expression in colon cancer cells [134].

5-FU is an antimetabolite used to treat various tumors. 5-FU incorporated into RNA severely damages RNA processing, especially ribosomal RNA, messenger RNA splicing. This modified deoxyribonucleotide also inhibits thymidylate synthase, causing thymine deficiency, resulting in cell cycle arrest and progression into apoptotic cell death. 5-FU causes cardiotoxicity and myelosuppression among other side effects in cancer patients. It is also demonstrated that resistance to 5-FU is dependent on JNK signaling [37].

It is worth mentioning that emergence of ROS during genotoxic stress is a very common feature in cancer therapy. As exemplified in a yeast model, many DNA-damaging agents increased intracellular ROS levels [135].

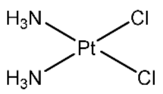

(a)

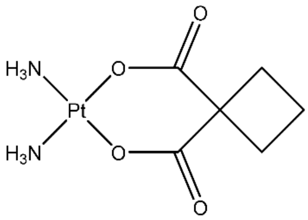

(b)

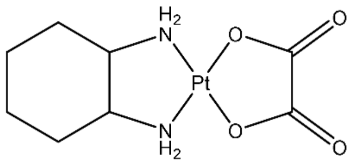

(c)

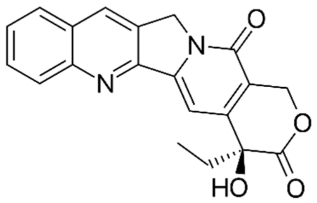

(d)

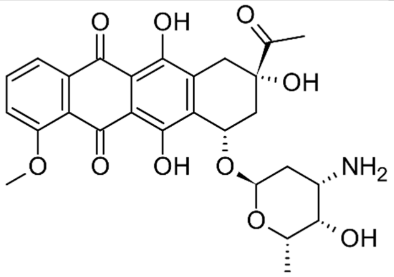

(e)

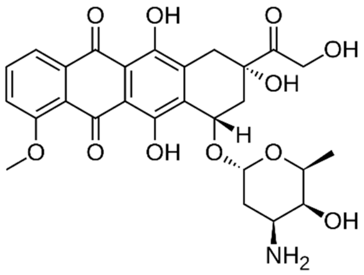

(f)

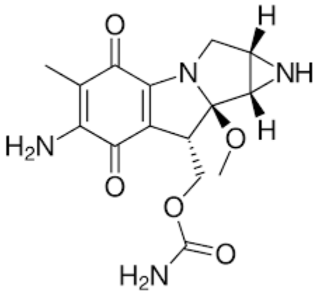

(g)

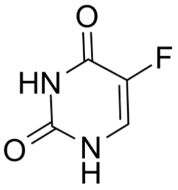

(h)

Figure S1. Chemical structures of DNA-damaging agents used in this study. (a) Cisplatin; (b) carboplatin; (c) oxaliplatin; (d) camptothecin; (e) daunorubicin; (f) doxorubicin; (g) mitomycin C; (h) 5-fluorouracil.

Inhibitor specificity

Supplementary Materials S2

Inhibitor selectivity notes are available at and taken from https://www.selleckchem.com/ :

SP600125: JNK1, JNK2 and JNK3 with IC50 of 40 nM, 40 nM and 90 nM in cell-free assays, respectively; 10-fold greater selectivity against MKK4, 25-fold greater selectivity against MKK3, MKK6, PKB, and PKCα, and 100-fold selectivity against ERK2, p38, Chk1, EGFR etc. This compound is also an inhibitor of Aurora kinase A, FLT3 and TRKA with IC50 of 60 nM, 90 nM and 70 nM.

RMC-6236: Daraxonrasib is a RAS(ON) multi-selective noncovalent inhibitor of the active, GTP-bound state of both mutant and wild-type variants of canonical RAS isoforms with broad therapeutic potential. This compound exhibits strong anticancer efficacy in RAS-addicted cell lines, especially those with mutations at codon 12 of KRAS.

KU60019: IC50 of 6.3 nM for ATM in cell-free assays, 270- and 1600-fold more selective for ATM than DNA-PK and ATR.

NAC: ROS (reactive oxygen species) inhibitor that antagonizes the activity of proteasome inhibitors. It is also a tumor necrosis factor production inhibitor. This compound suppresses TNF-induced NF-κB activation through inhibition of IκB kinases.

DMTU: noncompetitive inhibitor of the renal urea transporters UT-A1 and UT-B with an IC50 of 2-3 mM. It is also a scavenger of hydroxyl radicals, a reactive oxygen species (ROS).

Nutlin-3a: p53/MDM2 interaction with IC50 of 90 nM in a cell-free assay.

Capivasertib: IC50 of 3 nM, 8 nM and 8 nM for Akt1, Akt2 and Akt3, respectively; and ROCK2 56 nM.

AKT inhibitor VIII: Akt1/Akt2 inhibitor with IC50 of 58 nM/210 nM, respectively, about 36-fold selectivity for Akt1 over Akt3.

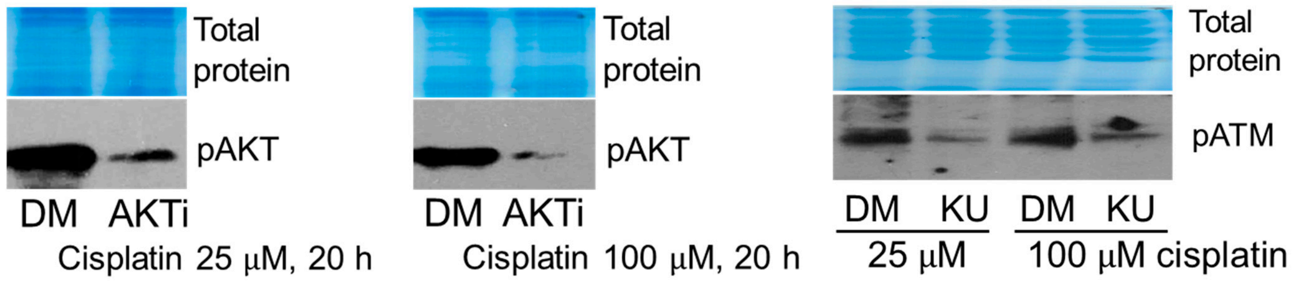

Figure S2. AKTi and KU60019 inhibit their target phosphorylation in cisplatin-treated A549 cells.

Apoptotic markers

Supplementary Materials S3

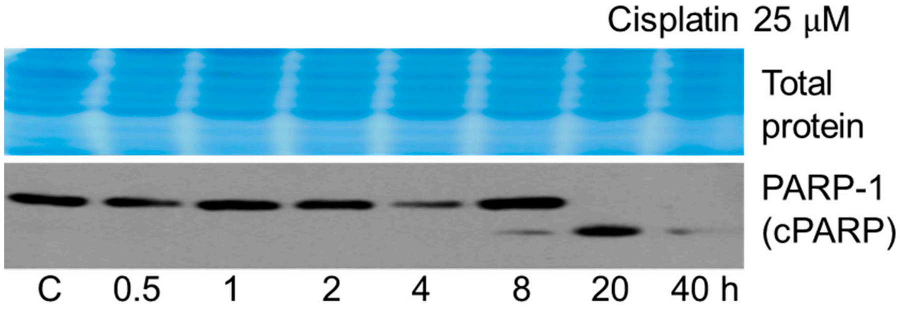

Figure S3. Cisplatin induces cleavage of PARP-1 protein, an early biochemical marker of apoptosis, in A549 cells.

Densitometry of Western blots

Supplementary Materials S4

The numbers above the bands indicate relative band intensity, after normalization to total protein loading control. ImageJ (v. 1.54g, from https://imagej.net/ij/download.html) software was used for measurements.

Figure 4 (in the main article):

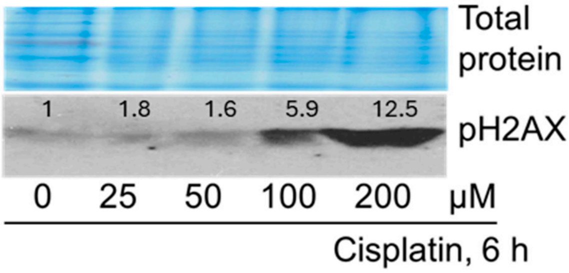

(a)

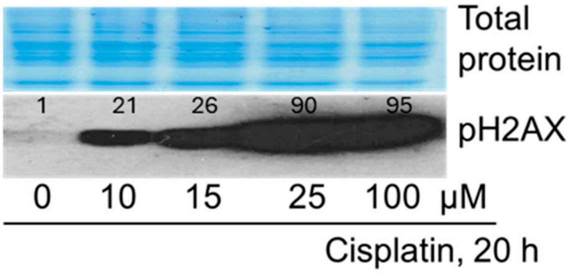

(b)

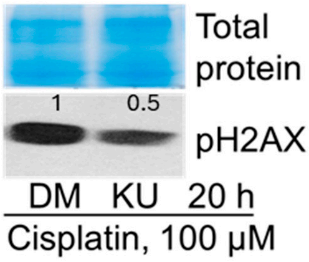

(c)

Figure 7:

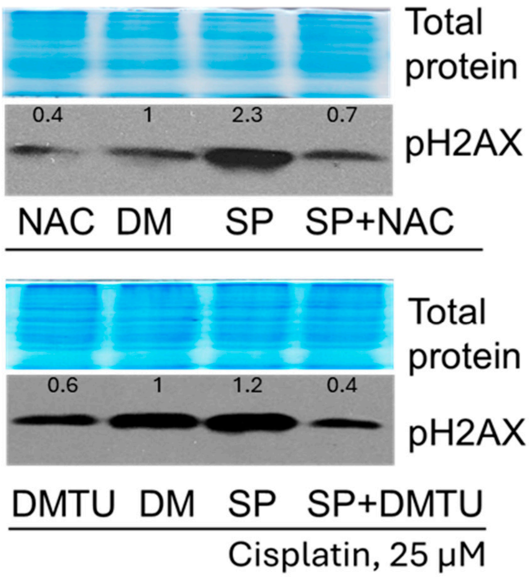

(a)

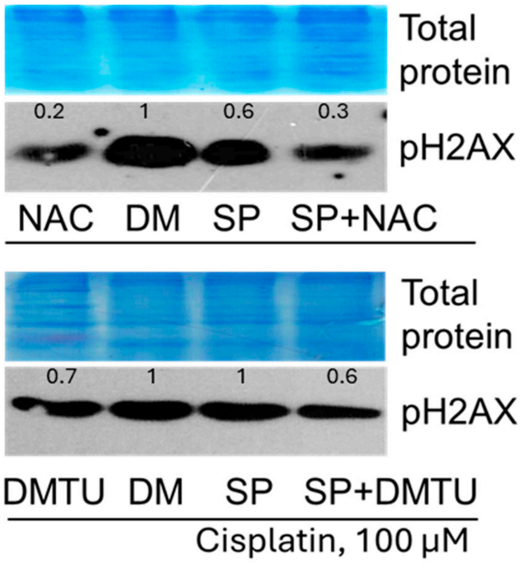

(b)

Figure 8:

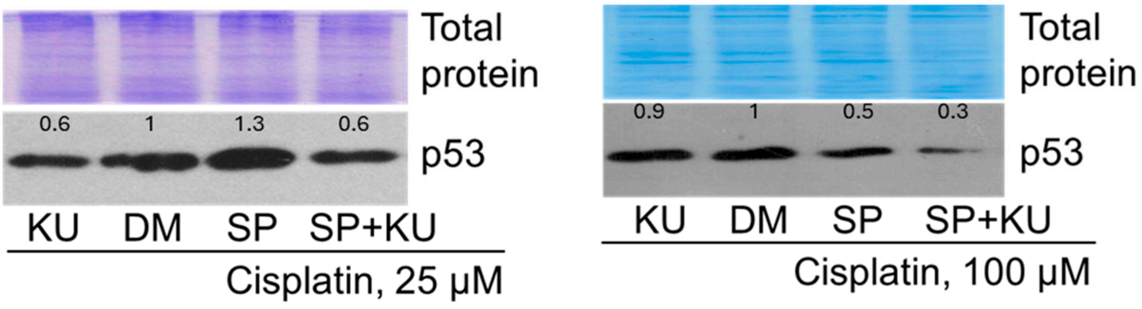

(a) **(b)**

Figure 9:

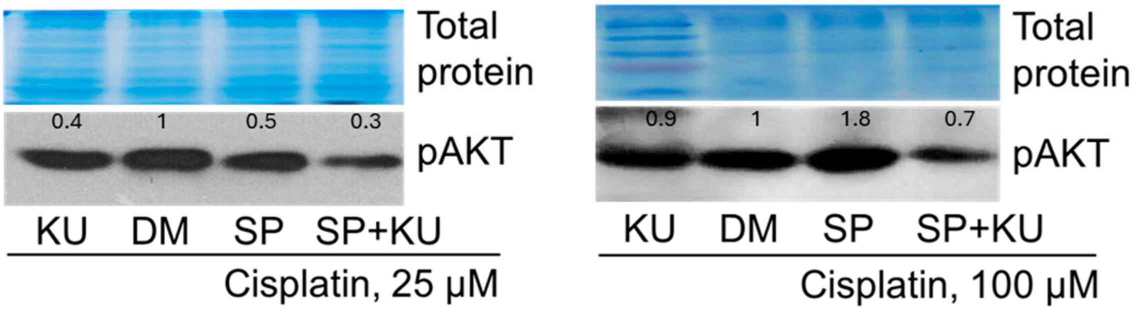

(a) **(b)**

Figure 10:

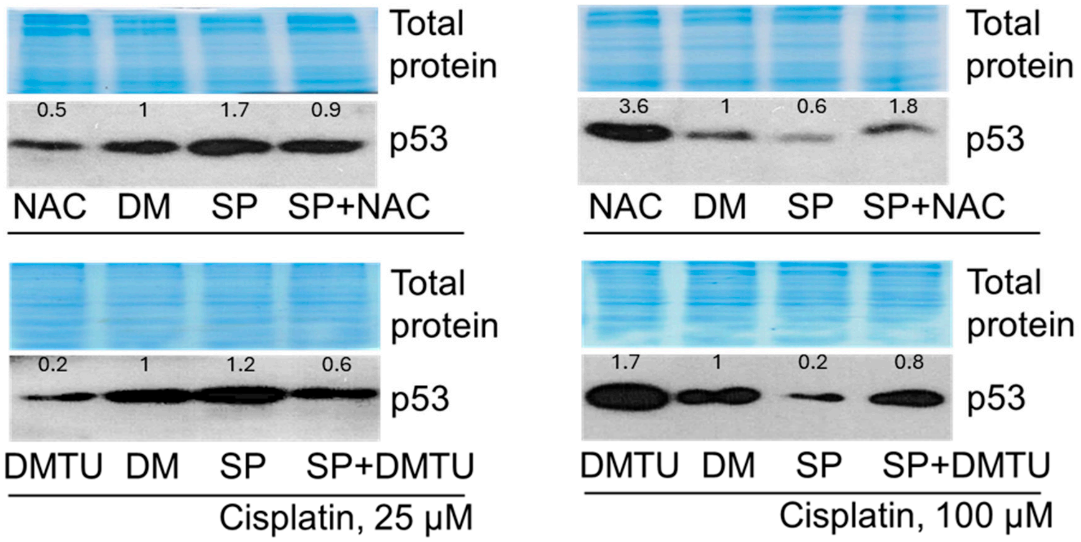

(a) **(b)**

Figure 11:

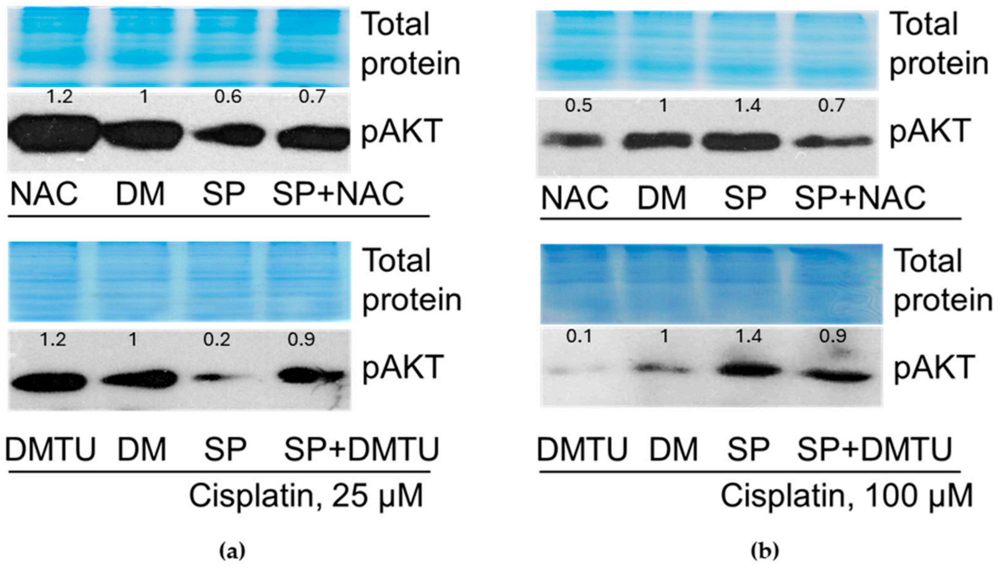

Figure 12:

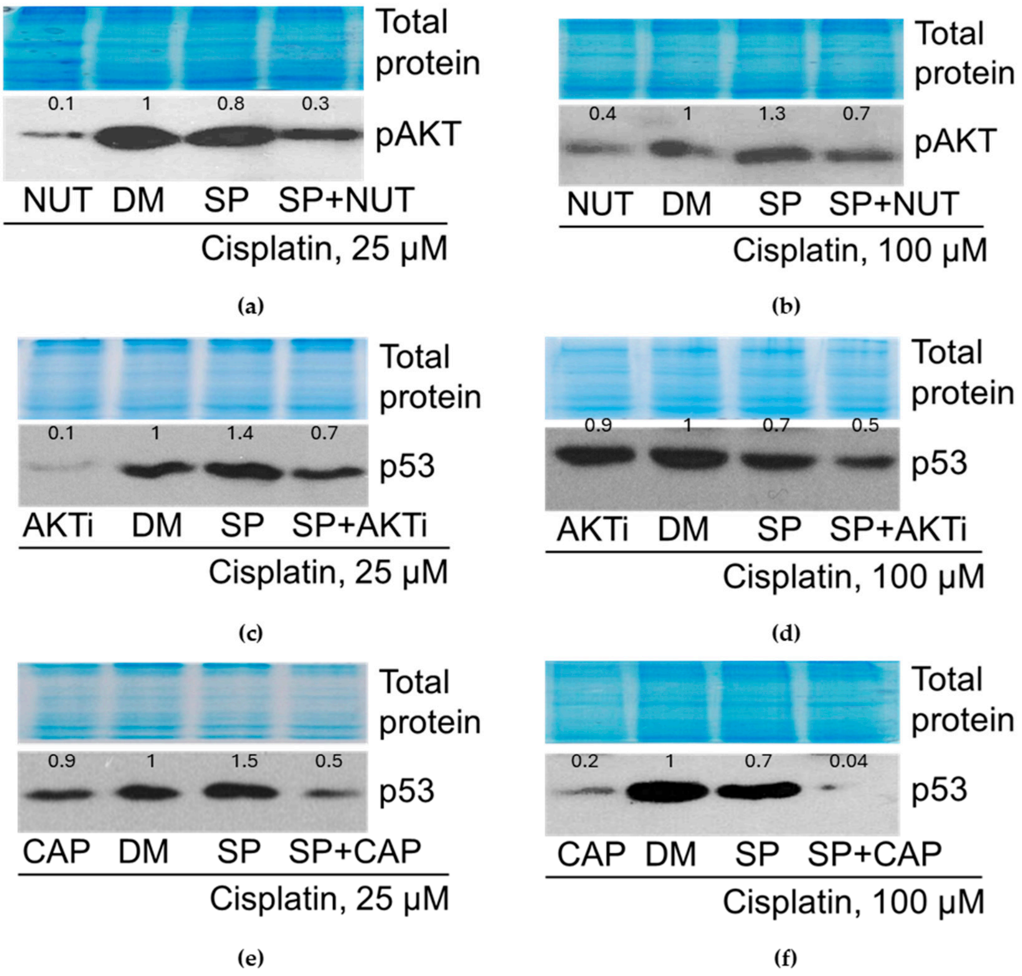

Supplement: Supplementary file 1 [file pharmaceuticals-19-00509-s001.zip › pharmaceuticals-4179448-supplementary.pdf]
